# The influence of extinction and counterconditioning procedures on operant evaluative conditioning and intersecting regularity effects

**DOI:** 10.1098/rsos.192085

**Published:** 2020-10-07

**Authors:** Sean Hughes, Simone Mattavelli, Ian Hussey, Jan De Houwer

**Affiliations:** 1 Department of Experimental Clinical and Health Psychology, Ghent University, Ghent, Belgium; 2 Department of Psychology, University of Milano-Bicocca, Milan, Italy

**Keywords:** intersecting regularities, extinction, counterconditioning, operant evaluative conditioning, implicit, attitudes

## Abstract

One of the most effective methods of influencing what people like and dislike is to expose them to systematic patterns (or ‘regularities’) in the environment, such as the repeated presentation of a single stimulus (mere exposure), two or more stimuli (evaluative conditioning (EC)) or to relationships between stimuli and behaviour (approach/avoidance). Hughes *et al*. (2016) *J. Exp. Psychol. Gen.* **145**, 731–754. (doi:10.1037/xge0000100) found that evaluations also emerge when regularities in the environment *intersect* with one another. In this paper, we examined if evaluations established via operant EC and intersecting regularities can be undermined via extinction or revised via counterconditioning. Across seven pre-registered studies (*n* = 1071), participants first completed a learning phase designed to establish novel evaluations followed by one of multiple forms of extinction or counterconditioning procedures designed to undo them. Results indicate that evaluations were—*in general*—resistant to extinction and counterconditioning. Theoretical and practical implications along with future directions are discussed.

## 1. Introduction

Over the past century, research in social and learning psychology has converged on a seemingly simple yet powerful idea: what we like and dislike is exquisitely sensitive to our interactions with the world around us. By exposing people to specific patterns of events in the environment (regularities), we can quickly and easily influence what they like and dislike.^1^

For instance, one can change liking by presenting the same stimulus over and over again: radio broadcasters often play a new song many times shortly after its release, and people repeatedly exposed to that song tend to evaluate it more positively than those who were not (i.e. the mere exposure (ME) effect; [3]). Another type of regularity involves pairing stimuli: advertisers often pair a neutral stimulus (e.g. a brand of perfume) with a valenced stimulus (e.g. images of a famous actress) to alter evaluations of the former in-line with the latter (i.e. evaluative conditioning (EC) effect; [4]). A third regularity involves relating certain actions to stimuli. For instance, the act of pushing alcohol away and pulling soft-drinks towards oneself influences evaluations of those stimuli as well as how much they are consumed (i.e. approach/avoidance (AA) effects; [5]). Although ME, EC and AA effects are all instances of evaluative learning, they differ in the type of regularity that leads to changes in liking (i.e. ME, regularity in the presence of one stimulus; EC, regularity in the presentation of two stimuli; AA: regularity between stimulus and action).

Yet, evaluative learning does not stop here. Hughes *et al*. [6] introduced another way of arranging the environment in order to influence evaluations. They labelled this procedure evaluative learning via *intersecting regularities* (IR). Whereas EC, ME and AA are relatively simple, insofar as they involve a change in liking owing to a single regularity (*see above*), IR procedures are more complex: they involve a situation where two or more regularities intersect with one another. By ‘intersect’, we mean that the regularities share one or more elements (e.g. a common stimulus or response), and because of this shared element, a change in liking occurs.

To illustrate this idea more clearly, consider the well-known sensory preconditioning procedure (see [2]). Here, two neutral stimuli (e.g. Bob and Chris) are initially paired with one another and one of the two is subsequently paired with an aversive stimulus (e.g. Bob is paired with unpleasant images). Research shows that people will come to dislike Bob *and* Chris even though Chris was never directly related with the unpleasant images. Such a procedure establishes *two* regularities between stimuli (i.e. one regularity involving the presentation of Bob and Chris; and another involving the presentation of Bob and unpleasant images). These two regularities also intersect in terms of a common element (Bob), and because of this intersection, a change in liking occurs (Chris is disliked). The dislike of Chris does not stem from a single regularity (e.g. Chris being paired with unpleasant images). Rather it stems from the intersection between one regularity (Bob–Chris) and another (Bob–unpleasant).

**Figure 1.**
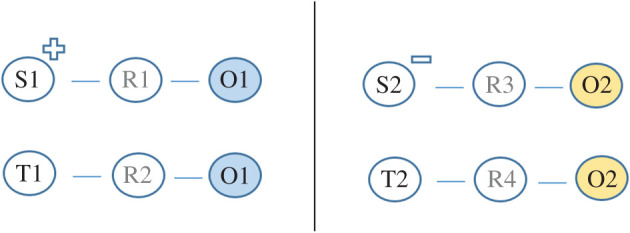
Schematic overview of the IR procedure from Hughes *et al*. [6] experiment 2. S refers to source stimulus, R to a response, O to an outcome stimulus and T to a target stimulus. The + and – indicate the valence of the source stimulus (either positive or negative).

Hughes *et al*. [6] argued that different regularities can be made to intersect with one another in many different ways, some of which have already been discovered (e.g. sensory preconditioning) and others that have not. To demonstrate their point, they had people complete a simple learning task wherein a certain button had to be pressed whenever a particular stimulus appeared onscreen (figure 1). For instance, if they pressed one button when a *positive* source stimulus was displayed then that stimulus disappeared and a neutral outcome stimulus took its place (positive source (S1) → response 1 → neutral outcome (O1)). If a neutral target appeared then pressing a second button caused that stimulus to disappear and the same neutral outcome to appear (neutral target (T1) → response 2 → neutral outcome (O1)). On other trials, pressing a third button whenever a *negative* source stimulus was on screen caused that stimulus to disappear and a second neutral outcome to take its place, while pressing a fourth button when a second neutral target was present caused the same neutral outcome to appear (i.e. negative source (S2) → response 3 → neutral outcome (O2); and neutral target (T2) → response 4 → neutral outcome (O2)).

Put simply, an operant contingency containing a valenced source stimulus ‘intersected’ with a contingency containing a neutral target stimulus (i.e. the two contingencies shared the same outcome stimulus). As a result, people liked target stimulus (T1) and disliked target stimulus (T2), even though neither was directly related with valenced source stimuli during the learning phase.^2^ These outcomes were obtained on self-reported, automatic, and behavioural intention measures (see [6] or [8], for demonstrations of various IR effects based on different types of operant contingencies; see [9] for a review and meta-analysis of studies on one type of IR effect; and see [6] for a discussion of real-world instances of IR effects).^3^

Until now, research on learning via IR has focused on how such procedures give rise to novel evaluative responses. Yet, the robustness of those evaluations still remains to be seen. In other words, can likes and dislikes established in this way be subsequently modified or eliminated using the procedures and methods commonly used to change evaluations using other regularities (such as stimulus pairing)? Given the applied and theoretical importance of research on the malleability of conditioned changes in liking, we deemed it important to examine the malleability of changes in liking that result from IR. In this paper, we examined the impact of two intervention procedures that have been highly popular in evaluative learning research: *extinction* and *counterconditioning*.

### 1.1. Extinction

Research on extinction typically relies on a procedure with two phases. Consider, for instance, extinction in the context of EC. In a first phase (acquisition), participants are exposed to a neutral conditioned stimulus (CS) which is paired with a valenced unconditioned stimulus (US). Thereafter, the valence of the CS typically changes in-line with that of the US. During the second phase (extinction), the CS is presented alone in the absence of the US. In this way, the extinction phase involves the removal of the (CS–US) contingency that originally gave rise to CS evaluations. Interestingly, many studies reveal no, or only a small, change in EC effects following an extinction procedure (e.g. [10–13]). That said, other studies have found that EC effects can be reduced following extinction trials [14,15]. A meta-analysis confirmed that, across studies, EC effects measured after the extinction procedure are smaller than those measured before an extinction procedure, although the former are still substantial [4]. These findings suggest that EC seems to be driven primarily by CS–US co-occurrences, rather than statistical contingency, and produces lasting changes in liking that persist even when CS and US no longer co-occur.

### 1.2. Counterconditioning

The robustness of evaluations can also be examined via counterconditioning. Similar to extinction, counterconditioning also tends to involve a procedure with two phases. For instance, during an initial (acquisition) phase, a contingency is established between two stimuli by pairing a neutral CS with a valenced US. In a second (counterconditioning) phase, the CS is then paired with a US of the opposite valence (e.g. a CS that was first paired with a positive is now paired with a negative US). People rate the CS in-line with the initial valence of the US after the first phase and then in-line with the subsequent valence of the US after the second phase (e.g. [16]).

### 1.3. The current research

Across a series of studies, we examined if evaluations established via IR or operant EC (see below) can be undone via extinction or modified via counterconditioning. This work was designed to explore environmental moderators of IR effects that proved to be vital in the study of other forms of evaluative learning.

#### 1.3.1. Examining the robustness of evaluations established via intersecting regularities

Our goal was to test if evaluations established via IR can be modified via extinction procedures (experiments 1–4) or counterconditioning procedures (experiments 5–7).

Experiments 1–3 sought to extinguish evaluations by removing the intersecting element (outcome stimulus) connecting source and target contingencies. We refer to this as an extinction-like procedure because, similar to extinction tasks in EC, it involves the removal of the environmental event that underlies the target evaluation (in this case, the common element shared by regularities).^4^ Because it proved difficult to consistently extinguish evaluations using such a task, we then decided (in experiment 4) to use an alternative procedure that has worked in the EC literature (non-contingent stimulus presentations). Once again, evaluations failed to extinguish. In experiment 5, we turned our attention to counterconditioning and attempted to do so by replacing the valenced source stimulus in one contingency with a stimulus of the opposite valence during the counterconditioning phase. Given the success of this manipulation, we then tried to countercondition evaluations, not by changing the valence of the source stimuli, but by ‘rearranging the intersection’ itself (i.e. experiments 6 and 7). Experiment 7 also tested the idea that there may have been a hidden intersection in our earlier studies that undermined the effectiveness of the extinction and counterconditioning manipulations.^5^

#### 1.3.2. Examining the robustness of operant evaluative conditioning effects

Although our primary goal was to test the robustness of intersecting regularity effects, our design also allowed us to explore a second issue. As noted earlier, the source contingencies in our studies (i.e. the operant contingencies that contained the valenced source stimulus) also included a neutral outcome. Consequently, the valence of the outcome stimulus could change in-line with the valence of the source stimulus. Whereas changes in liking of the *target* stimulus qualify as instances of IR effects (i.e. effects of intersections between regularities), changes in liking of the neutral *outcome* are instances of operant evaluative conditioning (OEC; i.e. effects of a single stimulus–action–outcome contingency; [17,18]). Put simply, OEC effects involve a change in liking that is owing to the relationship between stimuli and responses in an operant contingency. Our studies offered an opportunity to examine the formation, extinction and counterconditioning of OEC effects. As far as we know, this is the first time that extinction and counterconditioning of OEC has been examined.^6^

In all of our studies, we assessed liking via self-report ratings, the Implicit Association Test (IAT), and a behavioural intention task. We added the IAT because it is assumed to capture more automatic instances of evaluation. The behavioural intention task might reflect a more ecologically valid index of liking. Prior research on evaluative learning via IR has produced effects on each of these measures [6] and we expected similar outcomes here as well.

## 2. Experiments 1–4: extinction of operant evaluative conditioning and intersecting regularities effects

Our initial goal was to establish new likes and dislikes for outcome stimuli (OEC effect) and target stimuli (IR effects), and once these evaluations were in place, to eliminate them. We did so by removing the outcome stimulus from (i) the contingency containing the valenced source stimulus (experiment 1), (ii) the contingency containing the neutral target stimulus (experiment 2), or (iii) both contingencies (experiment 3). In experiment 4, we tried to degrade the intersection even more by presenting the target stimulus in isolation. This procedure not only eliminates intersections between contingencies but also highlights that the elements within those contingencies (stimuli and responses) are no longer related.

### 2.1. Method

#### 2.1.1. Participants and design

One hundred and forty-six participants (93 male, *M*_age_ = 27.9, s.d. = 5.3) (experiment 1), 108 participants (57 female, *M*_age_ = 29.7, s.d. = 7.2) (experiment 2), 111 participants (66 female, *M*_age_ = 28.8, s.d. = 5.8) (experiment 3) and 105 participants (54 male, *M*_age_ = 29.5, s.d. = 6.1) (experiment 4) completed the study on the Prolific website (https://prolific.ac) in exchange for a monetary reward.

A 2 (*stimulus*: neutral stimuli related to positive versus negative source) x 2 (*training*: extinction versus acquisition-only) mixed design was employed in experiments 1–4 with the first factor measured within and the second measured between participants. Self-reported ratings, IAT effects and behavioural intentions were the dependent variables. Three method factors were manipulated between participants: stimulus identity (whether outcome stimulus O1 and target stimulus T1 or outcome stimulus O2 and target stimulus T2 were assigned to positive/negative source stimuli), evaluative task order (self-report or IAT first) and IAT block order (learning consistent versus inconsistent block first).^7^

### 2.2. Materials

#### 2.2.1. Stimuli

Two fictitious brand names (Morag and Struan) and two Chinese ideographs served as neutral outcome and target stimuli, respectively, during the acquisition and extinction phases. These stimuli were selected based on a pre-test conducted on a different sample of 51 participants (17 women, *M*_age_ = 26.22, s.d. = 5.15), 47 of whom provided complete data and whose data were subsequently analysed. These participants were asked to evaluate two separate sets of 10 Chinese symbols and 10 fictitious brands by rating them on a scale from −5 to 5. The two selected Chinese ideographs were both neutral in valence: one sample *t*-tests indicated that their average score did not differ from 0, *t*_47_ = 0.67, *p* = 0.50 and *t*_47_ = 1.23, *p* = 0.23. A paired sample *t*-test indicated no differences in liking between the two, *t*_46_ = −0.33, *p* = 0.74. The two brand stimuli selected for use were the most neutral in valence, even though one did differ from 0, *t*_47_ = 2.63, *p* = 0.01, and *t*_47_ = 1.42, *p* = 0.16. Once again the two stimuli did not differ from one another in valence, *t*_46_ = 1.19, *p* = 0.24. A further set of 16 positive and 16 negative food images were used as valenced stimuli. In the IAT, two Chinese symbols from the learning phase served as target labels and the words ‘Good’ and ‘Bad’ as attribute labels. Eight positively valenced and eight negatively valenced adjectives served as attribute stimuli (*delicious*, *tasty*, *nice*, *good*, *gorgeous*, *wonderful*, *yummy* and *pleasant* versus *rotten*, *disgusting*, *nasty*, *horrid*, *sick*, *vomit*, *horrible* and *unpleasant*) while images of the two Chinese symbols served as target stimuli.

### 2.3. Procedure

**Figure 2.**
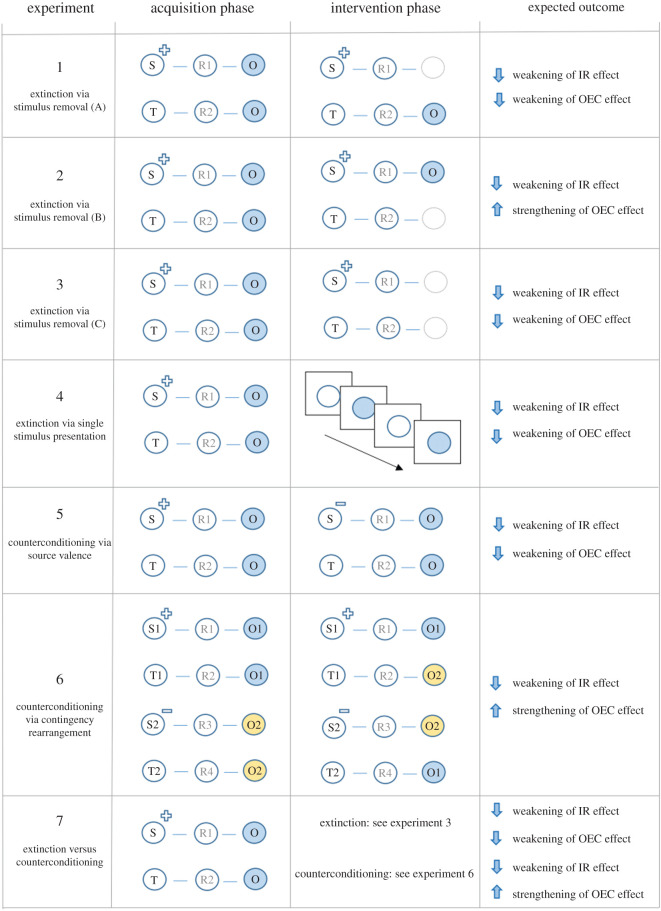
Schematic overview of the procedures and expected effects in experiments 1–7. S refers to source stimulus, R to a response, O to an outcome stimulus and T to a target stimulus. The + indicates a positive source stimulus and – indicates a negative source stimulus. For illustration purposes, figure 2 only displays one set of contingencies for most experiments (i.e. the ‘positively valenced’ contingencies, or those containing the blue outcome stimulus). However, each experiment also contained another set of ‘negatively valenced’ contingencies (e.g. see experiment 6 and the contingencies containing the yellow outcome stimulus).

Participants were provided with a general overview of the experiment, asked for their informed consent and then told that they would encounter a number of brand products that had purportedly been released into the European marketplace. One group (acquisition-only) completed an acquisition phase and then proceeded directly to the evaluative measures. The other (extinction) completed the acquisition followed by an extinction phase, and only then the evaluative measures. Everyone then answered a series of exploratory questions. The entire session took approximately 30 min. See figure 2 for an overview of the learning tasks used in experiments 1–7.

#### 2.3.1. Acquisition phase

##### 2.3.1.1. Training

Prior to the learning task, participants were informed that they would see an image (either food or a Chinese symbol) in the middle of the screen. Their task was to identify the specific key (either ‘D’, ‘C’, ‘J’ or ‘N’) that the item was related to. They were asked to take their time and try to be as accurate as possible. Training consisted of four blocks of 20 trials (80 total). Each trial began with the presentation of a positively or negatively valenced food image (i.e. source stimulus (S1) or (S2)) or one of two Chinese symbols (i.e. target stimulus (T1) or (T2)). Selecting (R1) in the presence of a positive source (S1) or (R2) when presented with neutral target (T1) resulted in the removal of that stimulus from the screen, followed by a 250 ms inter-stimulus interval and the subsequent presentation of a neutral brand name (i.e. outcome stimulus O1). After an inter-trial interval of 1250 ms, the next trial began. Likewise, selecting (R3) in the presence of a negative source (S2) or (R4) when presented with neutral target (T2) resulted in the removal of that stimulus from the screen, an inter-stimulus interval and the subsequent presentation of another brand name (outcome stimulus O2) (for an overview, see table 2). Stimulus-key assignments were counterbalanced between participants, such that one group categorized S1/T1 using R1/R2, whereas another group categorized S1/T1 using R3 and R4. If participants emitted an incorrect response then error feedback was displayed for 1500 ms. During this time, participants could not emit another response and had to wait until the next trial commenced in order to try again. Following each block, participants were exposed to a feedback screen that displayed their percentage accuracy during the previous section of the task. Instructions emphasized the need for accurate responding if past performance was below 90%.

##### 2.3.1.2. Testing

Following the training phase, a test block comprising eight trials was presented in order to examine if participants could report the stimulus–response and response–outcome relations (encountered during the training phase) in the absence of corrective feedback. The first four trials presented either a source or target stimulus, along with the four response options from the training phase and two other options (‘none of them’ and ‘I don't know’). Participants were asked to indicate what response had to be emitted when a given stimulus was presented. The next four trials presented a response option from the acquisition phase along with the two outcome stimuli, ‘neither of them’ and ‘I don't know’. Participants were asked to indicate what stimulus appeared when a given response was emitted. They then continued to the next phase of the experiment regardless of test performance.

#### 2.3.2. Extinction phase

##### 2.3.2.1. experiment 1

The extinction phase was similar to the acquisition phase (i.e. four blocks of 20 trials) with one exception. Once again, each trial began with the presentation of a positive (S1) or negative source (S2) or one of two neutral targets (T1 or T2). Selecting (R1) in the presence of a positive source (S1) resulted in the removal of that stimulus from the screen, but now, there was no subsequent presentation of an outcome. Selecting (R2) when presented with neutral target (T1) resulted in the removal of that stimulus from the screen followed by a 250 ms inter-stimulus interval, and the presentation of outcome (O1). After an inter-trial interval of 1250 ms, the next trial began. Selecting (R3) in the presence of a negative source (S2) resulted in the removal of that stimulus from the screen but no presentation of an outcome. Pressing (R4) when presented with neutral target (T2) resulted in the removal of that stimulus from the screen, an inter-stimulus interval and the presentation of outcome (O2) (figure 2). In the case of an incorrect response, an error feedback was displayed for 2000 ms. During this time, participants could not emit another response and had to wait until the next trial in order to try again. An identical test block to that presented after acquisition training was also presented after the extinction phase in experiments 1–3.

##### 2.3.2.2. experiment 2

The extinction phase was similar to that used in experiment 1 with one notable change. Whereas experiment 1 attempted to extinguish evaluative responding by removing the outcome from the contingencies containing the valenced sources, experiment 2 removed the outcome from the contingencies containing the neutral targets. Specifically, selecting (R1) in the presence of a positive source (S1) removed that stimulus from the screen, led to a 250 ms inter-stimulus interval and presentation of outcome (O1). Selecting (R2) when presented with neutral target (T1) removed that stimulus and was not followed by an outcome. Selecting (R3) in the presence of a negative source (S2) removed that stimulus from the screen, led to an inter-stimulus interval and presentation of outcome (O2). Pressing (R4) when presented with neutral target (T2) was not followed by an outcome (figure 2).^8^

##### 2.3.2.3. experiment 3

We now attempted to extinguish evaluative responding by removing the common intersection (outcome) from both contingencies. Selecting (R1) in the presence of a positive source (S1) or (R2) when presented with neutral target (T1) removed that stimulus from the screen, led to a 250 ms inter-stimulus interval and was not followed by an outcome. Selecting (R3) in the presence of a negative source (S2) or (R4) when presented with neutral target (T2) was also not followed by an outcome (figure 2).

##### 2.2.3.4. experiment 4

The extinction phase consisted of four blocks of 20 trials each. Participants were told that they would complete a second task wherein they would only have to observe a stream of stimuli. Each trial involved the presentation of a stimulus (T1, O1, T2, O2) for 1500 ms and each stimulus was presented five times per block. After an inter-trial interval of 1500 ms, the next trial began. No categorization response was required during this phase. Each type of stimulus was presented with equal frequency within each block. No testing block was also provided, given that no stimulus–response or response–outcome relations were encountered in this extinction procedure (figure 2).

### 2.4. Evaluative measures

#### 2.4.1. Implicit Association Test

An IAT was administered to measure relative automatic evaluations of the target stimuli. Participants were informed that the two Chinese symbols (T1 and T2) they had encountered during the learning phase (targets) as well as the words ‘Good’ and ‘Bad’ (attributes) would appear on the upper left and right sides of the screen. During each trial, a stimulus related to one of those categories would appear in the middle of the screen and they had to assign it to its corresponding category using either the left (E) or right keys (I). If they categorized the image or word correctly, the stimulus disappeared from the screen and the next trial began. By contrast, an incorrect response resulted in the presentation of a red ‘X’ which remained onscreen until the correct key was pressed.

Overall, each participant completed seven blocks of trials. The first block of 20 practice trials required them to sort the target stimuli into their respective categories, with one target (T1) assigned to the left (E) key and the other (T2) with the right (I) key. On the second block of 20 practice trials, participants assigned positively valenced stimuli to the ‘Good’ category using the left key and negative stimuli to the ‘Bad’ category using the right key. Blocks three (20 trials) and four (40 trials) involved a combined assignment of target and attribute stimuli to their respective categories. Specifically, participants categorized the first target (T1) and ‘positive’ words using the left key and the second target (T2) and ‘negative’ words using the right key. The fifth block of 20 trials reversed the key assignments, with target (T1) now assigned to the right key and target (T2) with the left key. The sixth (20 trials) and seventh blocks (40 trials) required participants to categorize target (T1) with ‘negative’ words and target (T2) with ‘positive’ words.

#### 2.4.2. Self-report measures

Ratings of the two outcome (brand names: O1 and O2) and target stimuli (Chinese symbols: T1 and T2) were obtained using a series of Likert scales. On each trial, participants were presented with a stimulus and asked to indicate whether they considered it to be ‘Good/Bad’, ‘Pleasant/Unpleasant’, ‘Positive/Negative’ and whether ‘I like it/I don't like it’ using a scale ranging from −5 to +5 with 0 as a neutral point.

#### 2.4.3. Behavioural intention task

This task comprised two trials: one trial in which the two target stimuli appeared simultaneously onscreen and another trial where the two outcome stimuli were presented. On the former trial, the stimuli appeared as labels on two bottles of ice-tea while on the latter trial, they appeared on two bottles of milk. Participants had to indicate, for each pair, which item they would choose if they encountered them in a supermarket. Five answers were possible (i.e. ‘I would choose product A’, ‘I would choose product B’, ‘I would choose both of them’, ‘I would choose neither of them’ or ‘I don't know’).

### 2.5. Exploratory questions

Participants completed *influence awareness*, *believability*, *demand compliance*, *reactance*, and *confidence* in their self-reported ratings measures. These latter questions were asked after the evaluative measures, were included for exploratory purposes and are therefore not mentioned in subsequent analyses.

### 2.6. Results

#### 2.6.1. Participant exclusions

We screened-out participants who (i) failed to complete the entire experimental session and thus provided incomplete data and/or (ii) who had IAT error rates above 30% across the entire task, above 40% for any one of the four critical blocks, or who completed more than 10% of trials faster than 400 ms (*n* = 49 (experiment 1), *n* = 14 (experiment 2), *n* = 16 (experiment 3) and *n* = 7 (experiment 4)). This led to a final sample of 97 participants in experiment 1, 94 in experiment 2, 95 in experiment 3 and 98 in experiment 4.

#### 2.6.2. Data preparation

Self-report ratings were collapsed into four mean scores—one for the target (T1), and another for the outcome (O1) related to positive sources, a third for the target (T2) and a fourth for the outcome (O2) related to negative sources. Two difference scores were then computed—one for the target stimuli (IR effect) and another for the outcome stimuli (OEC effect). Response latency data from the IAT were prepared using the D2 algorithm recommended by Greenwald *et al*. [19]. IAT scores reflect the difference in the mean response latency between the critical blocks divided by the overall variation in those latencies. Scores were calculated so that positive values reflected a preference for the target that was indirectly related to a positive source (T1) relative to that related to a negative source (T2). Negative values indicated the opposite.

#### 2.6.3. Analytic plan

We examined if behavioural intentions, self-reported and automatic stimulus evaluations (*dependent variables*) differed as a function of the type of training received (extinction versus acquisition-only) (*independent variable*). A series of *t*-tests were carried out on the rating and IAT data. With respect to the behavioural intentions data, only results from the T1–T2 comparison are reported (i.e. analyses were only carried out on responses that involved participants selecting either T1 or T2 and not on the selection of neither target, both targets or non-responses). Counts of each response for each study and experiment condition were calculated, which were then used to calculate an odds ratio. *p*-values were computed via Fisher's exact test. The Haldane–Anscombe corrections were applied to studies where at least one cell contained zero counts (i.e. counts in all cells were increased by 1).

### 2.7. Hypothesis testing

We focused on three questions. First, did participants demonstrate evidence of learning during the acquisition and extinction phases? If so, then they should respond with a high rate of accuracy (we labelled those who responded with greater than 75% accuracy during the final block of training or testing as having ‘passed’ that phase and those who did not as having ‘failed’).^9^ Second, did they demonstrate evidence of *evaluative* learning? If so then we would expect to observe an OEC effect (i.e. a preference for the outcome stimulus related to positive over negative sources) and an IR effect (i.e. a preference for the target stimulus related to positive over negative sources) when we examine the data from participants in the acquisition-only group. Third, did the extinction procedures implemented in experiments 1–4 undermine newly established evaluations? If so, then we would expect to observe a significant decrease in the OEC and IR effects relative to acquisition-only group.

#### 2.7.1. Question 1: how did participants perform during the acquisition and extinction phases?

As can be seen from table 1, participants responded with a high degree of accuracy during each phase of the learning task. The vast majority also met the necessary criterion to be labelled as having ‘passed’ a given phase of the learning task (table 2). One notable exception was the extinction testing phase in studies where the outcome stimulus was removed from both contingencies (experiments 3 and 7). This is despite the fact that those same participants had little difficulty passing the extinction training phase in those same experiments.

**Table 1.** Mean (and s.d.) accuracy as a function of learning task type (acquisition, extinction or counterconditioning training or testing) in experiments 1–7.

exp	acquisition training	acquisition test	extinction training	extinction testing	counterconditioning training	counterconditioning testing
1	88 (22)	85 (23)	96 (12)	82 (15)	/	/
2	93 (15)	88 (21)	98 (8)	89 (12)	/	/
3	91 (17)	83 (20)	97 (13)	61 (22)	/	/
4	93 (17)	85 (22)	/	/	/	/
5	93 (15)	87 (23)	/	/	97 (6)	87 (23)
6	95 (11)	90 (14)	/	/	98 (5)	90 (14)
7	89 (21)	80 (23)	97 (11)	75 (25)	97 (12)	93 (16)

**Table 2.** Percentage of participants who passed each section of the learning task (acquisition, extinction, counterconditioning) in experiments 1–7. (Counterconditioning was not provided in experiments 1–4 nor was extinction provided in experiments 5–6. The type of extinction procedure used in experiment 4 did not involve collection of training and testing data.)

exp	acquisition training	acquisition test	extinction training	extinction testing	counterconditioning training	counterconditioning testing
1	83	81	96	91	/	/
2	91	84	97	100	/	/
3	88	78	98	28	/	/
4	90	84	/	/	/	/
5	91	85	/	/	98	85
6	93	91	/	/	100	91
7	85	75	97	52	96	91

#### 2.7.2. Question 2: did evaluative learning take place?

##### 2.7.2.1. Operant evaluative conditioning effects

OEC effects emerged in all four studies. Participants self-reported that they liked O1 (the outcome that was part of a contingency with positive sources) and disliked O2 (the outcome that was part of a contingency with negative sources), experiment 1: *t*_47.93_ = 4.91, *p* < 0.0001, *d* = 1.31, 95% confidence interval (CI) (0.68, 1.93), Bayes Factor (BF)_10_ = 689; experiment 2: *t*_35.56_ = 5.85, *p* < 0.0001, *d* = 1.67, 95% CI (1.04, 2.3), BF_10_ > 10^4^; experiment 3: *t*_43.97_ = 6.08, *p* < 0.0001, *d* = 1.74, 95% CI (1.05, 2.43), BF_10_ > 10^4^; experiment 4: *t*_40.05_ = 6.24, *p* < 0.0001, *d* = 1.81, 95% CI (1.14, 2.49), BF_10_ > 10^4^. Likewise, the odds of selecting O1 were higher than those of selecting O2 in the behavioural intentions task: experiment 1 (odds ratio (OR) = 69.33, 95% CI (6.43, 748.06), *p* < 0.0001); experiment 2 (OR = 55.25, 95% CI (5.5, 555.07), *p* < 0.0001); experiment 3 (OR = 80, 95% CI (6.39, 1001.41), *p* < 0.0001); experiment 4 (OR = 13.22, 95% CI (1.4, 124.91), *p* = 0.01).

##### 2.7.2.2. Intersecting regularity effects

IR effects also emerged across studies. Participants self-reported that they liked T1 (the target that intersected with a contingency containing positive sources) and disliked T2 (the target that intersected with a contingency containing negative sources), experiment 1: *t*_47.55_ = 1.9, *p* = 0.06, *d* = 0.53, 95% CI (−0.05, 1.11), BF_10_ = 1.2; experiment 2: *t*_45.88_ = 2.61, *p* = 0.012, *d* = 0.72, 95% CI (0.16, 1.28), BF_10_ = 4.7; experiment 3: *t*_40.34_ = 5.68, *p* < 0.0001, *d* = 1.67, 95% CI (0.99, 2.35), BF_10_ > 10^4^; experiment 4: *t*_48.24_ = 3.03, *p* = 0.004, *d* = 0.83, 95% CI (0.23, 1.42), BF_10_ = 7.8. IAT scores demonstrated evidence for a relative preference for T1 over T2: experiment 1: *t*_44.43_ = 3.78, *p* < 0.001, *d* = 1.07, 95% CI (0.47, 1.67), BF_10_ = 75; experiment 2: *t*_50.19_ = 1.91, *p* = 0.06, d = 0.52, 95% CI (−0.03, 1.07), BF_10_ = 1.2; experiment 3: *t*_36.27_ = 4.6, *p* < 0.0001, *d* = 1.39, 95% CI (0.73, 2.04), BF_10_ = 856; experiment 4: *t*_39.6_ = 3.39, *p* = 0.002, *d* = 0.99, 95% CI (0.38, 1.59), BF_10_ = 29. Finally, the odds of selecting T1 were higher than those of selecting T2 in the behavioural intentions task in two of the four studies: experiment 1 (OR = 2.6, 95% CI (0.6; 11.31), *p* = 0.28); experiment 2 (OR = 15.12, 95% CI (2.28; 100.32), *p* = 0.003); experiment 3 (OR = 40, 95% CI (3.56; 450), *p* < 0.001); experiment 4 (OR = 2.89, 95% CI (0.69; 12.12), *p* = 0.17).

#### 2.7.3. Question 3: was evaluative learning moderated by the extinction procedures?

##### 2.7.3.1. Operant evaluative conditioning effects

Self-reported ratings decreased in magnitude (relative to the acquisition-only group) when the outcome stimulus was removed from both contingencies (experiment 3), *t*_92.88_ = −2.14, *p* = 0.04, *d* = −0.44, 95% CI (−0.85, −0.03), BF_10_ = 1.6. There was no difference between extinction and acquisition-only groups when the outcome was only removed from the source contingency (experiment 1): *t*_93.69_ = 0.87, *p* = 0.39, *d* = 0.18, 95% CI (−0.23, 0.58), BF_10_ = 0.3, or when stimuli were presented in a non-contingent manner (experiment 4), *t*_93.74_ = −1, *p* = 0.32, *d* = −0.2, 95% CI (−0.61, 0.2), BF_10_ = 0.3. As expected, OEC effects became stronger when the outcome stimulus remained in the source contingency and was removed from the target contingency (experiment 2): *t*_81.01_ = 4.48, *p* < 0.0001, *d* = 0.94, 95% CI (0.5, 1.38), BF_10_ = 922.

Behavioural intentions did not differ between the extinction and acquisition-only groups in experiment 1 (OR = 1.36, 95% CI (0.49, 3.76), *p* = 0.61), experiment 2 (OR = 0.55, 95% CI (0.19, 1.54), *p* = 0.30), experiment 3 (OR = 0.78, 95% CI (0.26, 2.31), *p* = 0.78) or experiment 4 (OR = 0.62, 95% CI (0.19, 2.1), *p* = 0.55).

##### 2.7.3.2. Intersecting regularity effects

No decrease in the magnitude of self-reported ratings (relative to the acquisition-only group) occurred when the outcome was removed from the source contingency (experiment 1): *t*_93.99_ = 1.38, *p* = 0.17, *d* = 0.28, 95% CI (−0.13, 0.69), BF_10_ = 0.5, both contingencies (experiment 3): *t*_92.82_ = −1.82, *p* = 0.07, *d* = −0.37, 95% CI (−0.78, 0.04), BF_10_ = 0.9 or when stimuli were presented in a non-contingent manner (experiment 4): *t*_90.04_ = 0.59, *p* = 0.56, *d* = 0.12, 95% CI (−0.28, 0.52), BF_10_ = 0.2. IR effects increased in magnitude when the outcome was removed only from the target contingency (experiment 2): *t*_74.64_ = 2.59, *p* = 0.012, *d* = 0.56, 95% CI (0.13, 0.98), BF_10_ = 4.6.

IAT scores also did not differ between the extinction and acquisition-only groups in experiment 1: *t*_85.72_ = −0.77, *p* = 0.44, *d* = −0.16, 95% CI (−0.56, 0.25), BF_10_ = 0.3; experiment 2: *t*_84.11_ = 0.46, *p* = 0.65, *d* = 0.1, 95% CI (−0.32, 0.51), BF_10_ = 0.2; experiment 3: *t*_90.64_ = −0.46, *p* = 0.65, *d* = −0.09, 95% CI (−0.5, 0.31), BF_10_ = 0.2; or experiment 4: *t*_91.22_ = −0.6, *p* = 0.5499, *d* = −0.12, 95% CI (−0.52, 0.28), BF_10_ = 0.3.

Finally, behavioural intentions did not differ between the extinction and acquisition-only groups in experiment 1: (OR = 0.82, 95% CI (0.28; 2.358), *p* = 0.79); experiment 2: (OR = 0.91, 95% CI (0.31; 2.68), *p* = 0.10); experiment 3: (OR = 0.52, 95% CI (0.17; 1.61), *p* = 0.28); experiment 4: (OR = 1.18, 95% CI (0.41; 3.37), *p* = 0.79).

### 2.8. Discussion

In experiments 1–4, people encountered an acquisition phase wherein an operant contingency containing a valenced source intersected with a contingency containing a neutral target (i.e. the two contingencies shared a common outcome stimulus). This phase was designed to establish novel evaluations towards outcome (OEC effect) and target stimuli (IR effect). Half of the participants then completed a second phase which removed the intersecting element from one contingency (experiments 1 and 2), both contingencies (experiment 3) or presented the stimuli in a non-contingent manner (experiment 4), to see if this would reduce or eliminate evaluations.

Results indicated that the acquisition phase gave rise to OEC and IR effects. However, we did not obtain evidence that the various ‘extinction’ procedures reduced or eliminated those evaluations. There was one exception: removing the outcome from both contingencies reduced OEC effects but this reduction was weak. The absence of extinction is particularly noteworthy, given the variety of procedures used, each of which eliminated the intersection present during the acquisition phase. Likewise, the absence of a reduced effect in experiment 4 is also noteworthy, given that this extinction procedure has been found to successfully extinguish EC effects (see [4], for a meta-analysis).

## 3. Experiments 5 and 6: counterconditioning

Given the difficulty of undoing evaluations established via operant evaluative conditioning and IR, we changed direction in experiments 5 and 6, and instead sought to revise likes and dislikes using counterconditioning procedures. Once again, participants completed an acquisition phase. Afterwards, one group moved directly to the evaluative measures while a second group first completed a counterconditioning task. In experiment 5, this involved replacing the valenced source stimulus in one operant contingency with a stimulus of the opposite valence. In experiment 6, this involved counterconditioning via ‘contingency rearrangement’ (see below).

### 3.1. Method

#### 3.1.1. Participants

One hundred and nine participants (69 male, *M*_age_ = 28.5, s.d. = 5.9) (experiment 5) and 106 participants (57 female, *M*_age_ = 29.7, s.d. = 5.6) (experiment 6) took part via Prolific Academic in exchange for a monetary reward.

### 3.2. Procedure

Overall, the study consisted of four phases: acquisition, counterconditioning, evaluative measures and exploratory questions. These phases were similar to those reported in experiments 1–4 unless otherwise stated.

#### 3.2.1. Counterconditioning

##### 3.2.1.1. experiment 5

The counterconditioning phase was similar to the acquisition phase with one notable exception: the assignment of valence source stimuli was reversed. Selecting (R1) in the presence of a negative source (S2), or (R2) when presented with neutral target (T1), resulted in the presentation of outcome (O1). Selecting (R3) in the presence of a positive source (S1), or (R4) in the presence of neutral target (T2), resulted in the presentation of outcome (O2) (figure 2).

##### 3.2.1.2. experiment 6

The counterconditioning procedure involved ‘contingency rearrangement’ and consisted of four blocks of 20 trials (80 trials total). Each trial began with the presentation of a positive (S1) or negative (S2) source, or a neutral target (T1 or T2). Selecting (R1) in the presence of a positive source (S1) removed it from the screen, produced a 250 ms intra-trial interval (ITI), and led to the presentation of outcome (O1). Selecting (R2) when presented with target stimulus (T1) resulted in its removal, an ITI and the presentation of outcome (O2). Selecting (R3) in the presence of a negative source (S2) resulted in its removal, an ITI and the presentation of outcome (O2). Selecting (R4) when presented with neutral target (T2) removed it from the screen and led to outcome (O1).

In short, we sought to rearrange the contingencies so that a ‘neutral’ contingency (T1 → R2 → O2) which previously intersected with a ‘positively valenced’ contingency (S1 → R1 → O1) now intersected with a ‘negatively valenced’ contingency (S2 → R3 → O2). We did the same with the other two contingencies (i.e. made a ‘neutral contingency’ that originally intersected with a ‘positively valenced contingency’ during acquisition now intersect with a ‘negatively valenced contingency’ during counterconditioning) (figure 2).^10^

### 3.3. Results

#### 3.3.1. Participant exclusions

Participants with incomplete data or who had excessive error or speed rates were excluded (*n* = 14 in experiment 5 and *n* = 16 in experiment 6). This resulted in a final *n* = 95 in experiment 5 and *n* = 90 in experiment 6.

#### 3.3.2. Hypothesis testing

We once again asked three questions. First, did participants learn the stimulus–response and response–outcome relations during the acquisition and counterconditioning phases? Second, did they demonstrate evidence of *evaluative* learning? Third, did the counterconditioning procedures undermine newly established evaluations? If so, we would expect a significant decrease in the magnitude of OEC and IR effects in the counterconditioning relative to acquisition-only group.

##### 3.3.2.1. Question 1: how did participants perform during the acquisition and counterconditioning phases?

As can be seen from table 1, participants responded with a high degree of accuracy during each phase of the learning task. The vast majority also met the necessary criterion to be labelled as having ‘passed’ a given phase of the learning task (table 2).

##### 3.3.2.2. Question 2: did evaluative learning take place?

*Operant Evaluative Conditioning effects*. OEC effects emerged in both studies. Participants self-reported that they liked O1 (the outcome that was part of a contingency with positive sources) and disliked O2 (the outcome that was part of a contingency with negative sources), experiment 5: *t*_42.86_ = 5.54, *p* < 0.0001, *d* = 1.64, 95% CI (0.95, 2.33), BF_10_ = 8148; experiment 6: *t*_35.56_ = 3.27, *p* = 0.002, *d* = 1.03, 95% CI (0.35, 1.71), BF_10_ = 15. The odds of selecting O1 were also higher than those of selecting O2 in the behavioural intentions task in experiment 5 (OR = 10.5, 95% CI (2.15, 51.28), *p* = 0.005) and experiment 6 (OR = 42, 95% CI (3.2, 551.57), *p* = 0.002).

*Intersecting regularity effects*. IR effects emerged in both studies. Participants self-reported that they liked T1 (the target that intersected with a contingency containing positive sources) and disliked T2 (the target that intersected with a contingency containing negative sources) in experiment 5: *t*_41.2_ = 3.15, *p* = 0.003, *d* = 0.94, 95% CI (0.31, 1.57), BF_10_ = 14; and experiment 6: *t*_39_ = 3.17, *p* = 0.003, *d* = 0.95, 95% CI (0.27, 1.62), BF_10_ = 9. IAT scores also demonstrated evidence for a relative preference for T1 over T2: experiment 5: *t*_37.19_ = 4.45, *p* < 0.0001, *d* = 1.35, 95% CI (0.69, 2.01), BF_10_ = 474; experiment 6: *t*_32.15_ = 3.61, *p* = 0.001, *d* = 1.17, 95% CI (0.47, 1.86), BF_10_ = 42. Finally, the odds of selecting T1 were higher than those of selecting T2 in the behavioural intentions task in experiment 5 (OR = 26.67, 95% CI (4.64; 153.22), *p* < 0.001), but not in experiment 6 (OR = 5.6, 95% CI (0.81; 38.51), *p* = 0.09).

##### 3.3.2.3. Question 3: was evaluative learning moderated by the counterconditioning procedures?

*Operant evaluative conditioning effects*. OEC as indexed by self-reported ratings decreased in magnitude (relative to the acquisition-only group) when counterconditioning involved reversing the valence of the source stimulus (experiment 5), *t*_85.69_ = −5.17, *p* < 0.0001, *d* = −1.07, 95% CI (−1.51, −0.63), BF_10_ = 12 450. It increased in magnitude, as expected, in the contingency rearrangement condition, which involved additional exposure to the OEC contingencies (experiment 6), *t*_86.47_ = 2.18, *p* = 0.032, *d* = 0.46, 95% CI (0.03, 0.89), BF_10_ = 1.7. Behavioural intentions did not differ between the counterconditioning and acquisition-only groups in experiment 5, OR = 1.6, 95% CI (0.57, 4.46), *p* = 0.44, or experiment 6, OR = 2.77, 95% CI (0.92, 8.32), *p* = 0.10.

*Intersecting regularity effects*. IR effects on self-reported ratings decreased in magnitude (relative to the acquisition-only group) when counterconditioning involved the reversal of source stimulus valence (experiment 5), *t*_64.79_ = −3.26, *p* = 0.002, *d* = −0.68, 95% CI (−1.1, −0.26), BF_10_ = 24, but not when contingency rearrangement took place (experiment 6), *t*_81.53_ = −0.84, *p* = 0.41, *d* = −0.18, 95% CI (−0.6, 0.24), BF_10_ = 0.3. IAT scores did not differ between the counterconditioning and acquisition-only groups in experiment 5 *t*_91.05_ = −1.87, *p* = 0.06, *d* = −0.39, 95% CI (−0.8, 0.03), BF_10_ = 1, or experiment 6: *t*_76.52_ = −0.85, *p* = 0.39, *d* = −0.18, 95% CI (−0.61, 0.24), BF_10_ = 0.3. Finally, behavioural intentions did not differ between the counterconditioning and acquisition-only groups in experiment 5: (OR = 1.26, 95% CI (0.43; 3.71), *p* = 0.79), or experiment 6: (OR=0.78, 95% CI (0.26; 2.34), *p* = 0.78).

### 3.4. Discussion

Experiments 5 and 6 exposed participants to an acquisition phase designed to establish novel evaluations towards outcome (OEC effect) and target stimuli (IR effect). Half of the participants then completed a second phase that sought to countercondition those evaluations via stimulus valence reversal (experiment 5) or contingency rearrangement (experiment 6). Results indicated that the acquisition phase gave rise to OEC and IR effects. Interestingly, whereas counterconditioning via stimulus reversal significantly decreased the OEC and IR effects on self-reported ratings (experiment 5), counterconditioning via contingency rearrangement only influenced OEC but not IR effects (experiment 6). When focusing on automatic preferences, neither counterconditioning via stimulus reversal nor counterconditioning via contingency rearrangement produced any change in the IR effect.

## 4. Experiment 7: extinction versus counterconditioning

In attempting to explain the resistance of IR effects to extinction and (to some extent) counterconditioning, we identified one possibility: many of the studies reported here involved not only a ‘visible’ intersection (the outcome) but also a ‘hidden’ intersection (response location). Specifically, during training, participants categorized one of the valenced sources and a neutral target using keys located on ‘left’ side of the keyboard (e.g. D or C). They also categorized the other valenced source and neutral target using keys on the ‘right’ side of the keyboard (e.g. J or N). Thus, stimuli not only intersected in terms of a common outcome but also in terms of a common response feature (use of left or right hand). This second intersection was still present during certain extinction phases (e.g. in experiments 1–3 but not in experiment 4 because responses were not made during the extinction phase of this experiment) and partially in experiment 5 (source stimulus mappings were reversed across the acquisition and counterconditioning phases) and experiment 6 (outcome stimulus mappings were reversed across acquisition to counterconditioning phases). Thus, even when certain outcome stimuli were no longer presented, and the intersection changed, participants often used the same hands to respond to S1 and T1 (left hand) and S2 and T2 (right hand). It may be that stronger extinction and counterconditioning effects emerge when both intersections (i.e. the outcome and the response location) are eliminated. We examined this possibility in experiment 7. We were also interested in comparing the relative effectiveness of extinction or counterconditioning in changing IR effects. We, therefore, recruited three groups of participants and exposed them to either (i) only the acquisition phase, (ii) acquisition and then extinction, or (iii) acquisition and then counterconditioning.

### 4.1. Method

#### 4.1.1. Participants and design

Three hundred and eighty-six participants (222 women, *M*_age_ = 29.1, s.d. = 5.8) took part in an online experiment via Prolific Academic in exchange for a monetary reward.

### 4.2. Procedure

Participants completed an acquisition phase, and either proceeded to the evaluative measures (acquisition-only) or first completed an extinction or counterconditioning task.

#### 4.2.1. Acquisition phase

The structure of the acquisition phase was similar to that administered in experiments 1–6 with two exceptions: participants now emitted a response using a mouse rather than keyboard and the location of the responses varied randomly across trials (thereby ensuring no common response location could emerge). The four response options (D, C, J and N) were printed onscreen below the stimulus on each trial. Clicking on one of the four letters with the mouse led to the removal of the stimulus, a short (250 ms) intra-trial interval, and finally the outcome stimulus. Pilot testing indicated that participants found this version of the task to be difficult. We, therefore, provided a fifth block of trials in situations where they emitted less than 80% correct responses during the fourth training block.

#### 4.2.2. Extinction phase

A similar extinction phase was used as in experiment 3 with three exceptions: we changed the nature of responding (mouse instead of key-press), randomized the location of response options across trials and provided a fifth block of trials for participants who emitted less than 80% correct responses during the fourth block of training.

#### 4.2.3. Counterconditioning phase

A similar counterconditioning phase was used as in experiment 6 with two exceptions: we changed the nature of responding (mouse instead of key-press), randomized the location of response options across trials and provided a fifth block of trials for participants who emitted less than 80% correct responses during the fourth block. Once again, this counterconditioning phase was expected to reduce IR effects and boost OEC effects.

#### 4.2.4. Exploratory questions

Along with the other questions, we also included a matching to sample procedure. This task was included for exploratory purposes, delivered at the very end of the experiment and will not be discussed further.

### 4.3. Results

#### 4.3.1. Participant exclusions

Participants with incomplete data or who had excessive IAT error or speed rates were excluded (*n* = 73). This led to a final sample of 313 participants.

#### 4.3.2. Hypothesis testing

We were interested in four questions. First, did participants learn the stimulus–response and response–outcome relations during the acquisition and intervention phases? Second, did they demonstrate evidence of *evaluative* learning? Third, did the extinction and/or counterconditioning procedures undermine newly established evaluations? Fourth, was counterconditioning or extinction more effective in doing so?

##### 4.3.2.1. Question 1: how did participants perform during the acquisition and intervention phases?

As can be seen from table 1, participants responded with a high degree of accuracy during each phase of the learning task. Most also met the criterion needed to be labelled as having ‘passed’ a given phase of the learning task (table 2).

##### 4.3.2.2. Question 2: did evaluative learning take place?

*Operant evaluative conditioning effects*. OEC effects emerged such that participants self-reported liking O1 and disliking O2: *t*_98.77_ = 5.43, *p* < 0.0001, *d* = 1.06, 95% CI (0.65, 1.48), BF_10_ = 33 252. Behavioural intentions also favoured O1 over O2, OR = 7.56, 95% CI (2.26, 25.22), *p* < 0.001.

*Intersecting regularity effects*. IR effects emerged such that participants self-reported liking T1 and disliking T2: *t*_98.37_ = 2.24, *p* = 0.028, *d* = 0.44, 95% CI (0.05, 0.83), BF_10_ = 1.9. IAT scores also demonstrated evidence of a relative preference for T1 over T2, *t*_101.79_ = 3.92, *p* < 0.001, *d* = 0.77, 95% CI (0.37, 1.17), BF_10_ = 146. Behavioural intentions did not favour T1 over T2, OR=2, 95% CI (0.69, 5.76), *p* = 0.29.

##### 4.3.2.3. Question 3: was evaluative learning moderated by extinction or counterconditioning?

*Operant evaluative conditioning effects*. OEC effects did not decrease in magnitude relative to the acquisition-only group when the outcome was removed from both contingencies (extinction), *t*_187.92_ = 0.69, *p* = 0.49, *d* = 0.1, 95% CI (−0.18, 0.37), BF_10_ = 0.2. However, they increased, as expected, following counterconditioning, which involved additional exposure to operant evaluative conditioning, *t*_204.38_ = 2.23, *p* = 0.03, *d* = 0.31, 95% CI (0.03, 0.58), BF10 = 1.5. Evidence did not emerge to support the idea that behavioural intentions were moderated by either the extinction, OR = 0.9, 95% CI (0.43, 1.88), *p* = 0.85, or counterconditioning procedures, OR = 0.61, 95% CI (0.3, 1.23), *p* = 0.21.

*Intersecting regularity effects*. Neither IR effects indexed by self-reported ratings, *t*_202.2_ = 1.56, *p* = 0.12, *d* = 0.22, 95% CI (−0.06, 0.49), BF_10_ = 0.5, IAT scores, *t*_205.09_ = 1.11, *p* = 0.27, *d* = 0.15, 95% CI (−0.12, 0.43), BF_10_ = 0.3, nor behavioural intentions, OR = 1.1, 95% CI (0.53, 2.29), *p* = 0.85, differed in the extinction relative to acquisition-only group. Although IR effects as indexed by self-reports decreased in the counterconditioning (relative to acquisition-only) group, *t*_207_ = −2.5, *p* = 0.01, *d* = −0.35, 95% CI (−0.62, −0.07), BF_10_ = 2.7, this was not the case for IAT scores, *t*_206.02_ = 0.84, *p* = 0.40, *d* = 0.12, 95% CI (−0.16, 0.39), BF_10_ = 0.2, nor behavioural intentions, OR=1.24, 95% CI (0.61, 2.53), *p* = 0.59.

##### 4.3.2.4. Question 4: which was more effective in moderating evaluations: extinction or counterconditioning?

A series of paired *t*-tests showed that IR effects as indexed by self-report ratings were smaller after counterconditioning than after extinction, *t*_203.14_ = −3.91, *p* < 0.001, *d* = −0.54, 95% CI (−0.82, −0.26), BF_10_ = 167. This difference was not found for IR effects as indexed by IAT, scores, *t*_203.3_ = −0.34, *p* = 0.73, *d* = −0.05, 95% CI (−0.32, 0.23), BF_10_ = 0.2 or behavioural intentions, OR = 1.13, 95% CI (0.56, 2.29), *p* = 0.86, nor for OEC effects as indexed by self-reported ratings, *t*_198.46_ = 1.24, *p* = 0.22, *d* = 0.17, 95% CI (−0.1, 0.45), BF_10_ = 0.3 or behavioural intentions, OR = 0.67, 95% CI (0.34, 1.33), *p* = 0.29.

### 4.4. Discussion

Once again, OEC and IR effects emerged. An extinction procedure which removed the outcome stimulus from both contingencies did not influence the magnitude of these newly established evaluations. Likewise, a counterconditioning procedure which involved contingency rearrangement was only partially successful in that it reduced IR effects as indexed by self-report, but not IAT scores or behavioural intentions. Directly comparing the impact of the extinction and counterconditioning procedures revealed that the latter decreased self-reported evaluations (but not IAT scores or behavioural intentions) to a greater extent than the former.

## 5. Meta-analyses

We carried out a series of multilevel meta-analyses to ask three general questions about our findings that individual studies lacked the power to address or to make general conclusions from: (i) do OEC and IR procedures give rise to evaluations *in general*, (ii) are evaluations moderated by extinction or counterconditioning *in general*, and (iii) do those effects differ when we exclude participants who failed the learning task? Analyses were conducted using the metafor R package [20]. All models employed a restricted maximum-likelihood estimator function. In each case, study was entered as a random intercept in order to acknowledge the non-independence of each study's outcome variables, and outcome variable type (i.e. IAT, self-reported evaluations, behavioural intentions) was entered as a random slope in order to acknowledge that changes of different magnitudes may be observed between them. Prior to meta-analysis, behavioural intention data were converted from odds ratios to Cohen's *d* scores using the method specified by Hasselblad & Hedges ([21]; see also [22]) which has been shown to balance ease of use, bias and coverage. Meta-analyses were not pre-registered, although the hypotheses assessed within them were similar to those pre-registered in the individual experiments.

### 5.1. Question 1: Do operant evaluative conditioning and intersecting regularities procedures give rise to novel evaluations in general?

**Figure 3.**
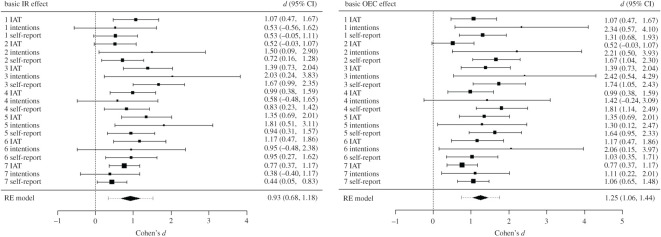
Meta-analytic models outlining the IR and OEC effects. In each forest plot, squares represent observed Cohen's *d* effect sizes, size of square represents weighting in the model and error bars represent 95% confidence intervals (CIs) around the effect size.

Each of our studies employed multiple evaluative measures (self-reports, IATs, behavioural intentions). These measures were not included for theoretical reasons (e.g. to examine dissociations between automatic and non-automatic evaluations) but instead to provide convergent evidence for evaluative learning. We, therefore, wanted to know if OEC and IR gave rise to novel evaluations *in general* (i.e. regardless of the specific measure used). To answer this question, we carried out multilevel meta-analyses of both the IR and OEC effects within the acquisition-only group (figure 3).

#### 5.1.1. Operant evaluative conditioning effects

The meta-analytic model indicated that a change in liking takes place after OEC, *d* = 1.25, 95% CI (1.06, 1.44), *p* < 0.001.

#### 5.1.2. Intersecting regularity effects

The meta-analytic model indicated that a change in liking takes place after IR training, *d* = 0.93, 95% CI (0.68, 1.18), *p* < 0.001.

### 5.2. Question 2: Are operant evaluative conditioning and intersecting regularities effects moderated by extinction or counterconditioning?

**Figure 4.**
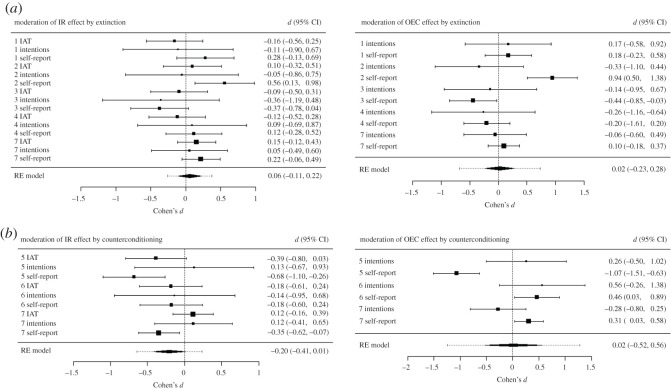
Meta-analytic models outlining the moderation of the IR and OEC effects by intervention type (extinction (*a*) or counterconditioning (*b*)). In each forest plot, squares represent observed Cohen's *d* effect sizes, size of square represents weighting in the model and error bars represent 95% confidence intervals (CIs) around the effect size. The *d* score in the above figure indicates a difference between the acquisition-only and acquisition plus intervention conditions, where positive scores indicate that the effect was strengthened by the intervention whereas negative scores indicate that it was weakened.

Four variants of extinction procedure and two counterconditioning procedures were implemented in experiments 1–7. These interventions moderated evaluations in certain studies and failed to do so in others. The question remains: to what extent do ‘extinction’ and ‘counterconditioning’ moderate evaluations that were established via IR *in general*? A multilevel meta-analysis was conducted on the OEC and IR effects to answer this question. It is worth reiterating that the extinction and counterconditioning procedures were primarily designed to modify IR effects. In certain cases (experiments 2, 6, 7), these procedures boosted rather than undermined OEC effects. As such, the meta-analytic effect for the OEC effects should be treated with caution, and the forest plot is only provided as a visual overview of OEC effects across studies (figure 4).

#### 5.2.1. Extinction

The meta-analytic model indicated that, in general, there was no evidence to support the idea that OEC effects, *d* = 0.02, 95% CI (−0.23, 0.28), *p* = 0.85, nor IR effects, *d* = 0.06, 95% CI (−0.11, 0.22), *p* = 0.49, were moderated by the extinction procedures used in this paper.

#### 5.2.2. Counterconditioning

The meta-analytic model indicated that, in general, there was no strong evidence to support the idea that IR effects were moderated by the counterconditioning procedures used in this paper, *d* = −0.20, 95% CI (−0.41, 0.01), *p* = 0.06.

### 5.3. Question 3: do our conclusions change when only considering those who passed the learning phase?

So far, we have analysed the data of all participants regardless of their performance on the learning task. However, upon reflection, people who performed poorly during that task may be responsible for the absence of extinction and counterconditioning (i.e. if they did not discriminate the contingencies during the acquisition and intervention phases then it seems unlikely that evaluative effects will emerge or be later modified). We, therefore, carried out a similar set of analyses as reported above, but exclusively on the data from the ‘pass’ group (i.e. people who demonstrated accuracy greater than 75% on the final block of training and testing in the learning task). Afterwards, a series of robustness checks were carried out to investigate if the conclusions derived from the entire sample were congruent or incongruent with those derived from the pass group. These analyses indicated that conclusions regarding (i) the significance of IR and OEC effects, (ii) moderation by extinction, and (iii) moderation by counterconditioning were congruent between the meta-analysis of the entire data and those of the pass group data (see the electronic supplementary material). Thus, the absence of extinction and counterconditioning effects in the entire sample cannot be attributed to a failure of participants to ‘learn’ during the acquisition and intervention phases.

## 6. General discussion

Across seven studies, we sought to gain a deeper understanding of the conditions under which evaluations established via IR or OEC can either be undone (via extinction) or modified (via counterconditioning). During an acquisition phase, participants learned that a contingency containing a valenced source ‘intersected’ with a contingency containing a neutral target (i.e. that they both contained a common outcome stimulus). An extinction procedure was then administered which eliminated the intersection by removing the common outcome from the valenced (experiment 1), target (experiment 2) or both contingencies (experiment 3). Experiment 4 examined if a different extinction procedure (CS-only presentations) would eliminate evaluations. In experiments 5–7, we sought to countercondition evaluations, by either replacing the valenced source with a stimulus of the opposite valence (experiment 5) or by contingency rearrangement (experiments 6 and 7). Participants in the acquisition-only group never encountered an extinction or counterconditioning phase and proceeded directly to the evaluative measures.

### 6.1. Summary of findings

#### 6.1.1. Intersecting regularities

A multilevel meta-analysis of experiments 1–7 shows that evaluative learning via IR gives rise to strong changes in likes and dislikes, replicating prior work in this area [6]. Meta-analyses also indicated that—*in general*—there was little evidence to support the idea that the extinction procedures used in this paper led to a reduction in IR or OEC effects, or that the counterconditioning procedures led to a reduction in IR effects.

#### 6.1.2. Operant evaluative conditioning

A multilevel meta-analysis of experiments 1–7 also showed that OEC gave rise to strong changes in likes and dislikes. Meta-analyses also indicated that—*in general*—there was little evidence to support the idea that the extinction procedures used in this study reduced OEC effects. By contrast, in experiment 5, the only study designed to countercondition OEC effects, self-reported ratings were reduced when source stimulus valence was reversed from acquisition to counterconditioning.

### 6.2. Empirical implications

#### 6.2.1. Extinction of evaluations

On the one hand, our findings are broadly consistent with past work, suggesting that evaluations established via regularities (e.g. EC) can be difficult to extinguish ([4]; but see [14,15]). It seems that once a relationship between source and target stimuli has been established, and the valence of the former has transferred to the latter, removing the intersection that initially gave rise to those evaluations may be ‘too little, too late’ (i.e. post-acquisition changes to the intersection does not decrease liking).

On the other hand, the absence of extinction effects could have been owing to the specific parameters used in our studies and extinction may occur if other conditions are met. For instance, it may be that participants viewed the contingencies during the acquisition phase as being a-contextual and the altered contingencies they encountered during the extinction phase in a highly contextual manner (i.e. what was initially learned (acquisition) applies across contexts whereas what is later learned (extinction) only applies to one specific context; for related work, see [23]). Likewise, although we eliminated the regularity during the extinction phase, the valenced stimulus was often still present, a factor that could also have contributed to the persistence of the effect. It is also possible that extinction of evaluations could be facilitated by using a single instead of multiple valenced sources (as we used), presenting stimuli simultaneously instead of sequentially, or even asking participants to rate the targets and outcomes multiple times. Future work should better study the boundary conditions of extinction in the context of IR and OEC (for one such example, see [24], experiment 2).

#### 6.2.2. Counterconditioning of evaluations

Our findings also suggest that IR effects might be difficult to countercondition. This finding is surprising in that other types of evaluative learning are sensitive to counterconditioning procedures. For instance, when it comes to EC, preferences can be reversed or be eliminated following experience [25] or instruction-based counterconditioning via stimulus valence reversal [12], and the former is often more effective than the latter [25]. In the impression formation literature, evaluations can be formed when people are told that certain positive behaviours are characteristic of a fictional person and then later reversed when they are given contradictory information (e.g. [26]). Moreover, counterconditioning seems to be a more powerful technique for changing evaluations than other procedures such as extinction. This is true not only for likes and dislikes [12], but also fear [27], disgust [28] and eating behaviours [29]. It is, therefore, surprising that we failed to obtain strong evidence of counterconditioning in our own studies. Looking to the future, we see several possibilities. Reversing the valence of the source stimulus in experiment 5 impacted self-reported ratings and IAT scores more than the contingency rearrangement approach used in experiments 6 and 7, suggesting that the former might be a more promising avenue to pursue than the latter. Future work could attempt to replicate our finding that source valence counterconditioning alters IR effects, examine if still other counterconditioning procedures might be more effective than those used here (e.g. instruction-based variants) and whether counterconditioning is more or less effective than other evaluative change procedures (e.g. US revaluation).

### 6.3. Theoretical implications

Although our studies were designed primarily with the aim to explore the malleability of evaluative learning via IR, our findings do impose constraints on mental theories of evaluative learning, insofar as these theories have to explain why evaluations established via IR or OEC are resistant to extinction but sensitive to counterconditioning. We consider two types of mental models: associative and propositional perspectives.

#### 6.3.1. Associative (mental) models

Associative models refer to a class of models that each share the idea that evaluative learning is mediated by associations between mental representations. These models differ in the specific assumptions they make about the formation and nature of those associations (e.g. unidirectional versus bidirectional associations). Although it is impossible to prove or disprove such a broad class of models, our results do place further constraints on them. Associative models could assume a chain of associations via which the evaluation of the source can spread to the evaluation of the target. For instance, when pressing R1 (e.g. D key) in response to S1 (positive foods) leads to O1 (first Chinese symbol), a direct association might be formed between S1 and R1, and between R1 and O1, while an indirect association is formed between S1 and O1. Likewise, when pressing R2 (e.g. C key) in response to T1 (first brand name) leads to O1, direct associations might form between T1–R2, R2–O1 and an indirect association between T1–O1. Hence, a positive evaluation of T1 might arise if T1 activates O1 (via R2 or directly) and if O1 leads to the activation of the positive valence of S1 (via R1 or directly). Note that such an account already constrains associative models beyond the constrains enforced by evaluative conditioning effects because it implies that activation can spread across a chain of associations not only in a forwards (e.g. T1 activates O1) but also in a backwards direction (e.g. O1 activates S1). The latter assumption is not trivial as it is often assumed that activation can only spread in a forwards manner across associations (e.g. [30]).

An alternative way for associative models to deal with the IR effects reported in this paper is to assume that the outcomes acquire an intrinsically positive or negative valence as a result of the S–R–O trials. This valence can then transfer to the targets on T–R–O trials. The crucial difference with the associative account put forward in the previous section is that evaluative responses (i.e. valence) become associated directly with outcome and target stimuli (i.e. stimulus–response associations) without having to assume associations between stimulus representations (i.e. stimulus–stimulus associations; see [31]). For instance, once O1 evokes positive responses as the result of S1(positive)–R1–O1 trials, those positive responses could become associated with T1 as the result of T1–R2–O1 trials. It should be noted, however, that associative models that assume the formation of stimulus–response associations fail to account for other key findings in the evaluative learning literature (e.g. US revaluation; see [4], for a review). Moreover, in order to account for the current data, such models need to allow for the formation of stimulus–response associations independently of the order in which stimuli appear (e.g. both when the positive S1 precedes the initially neutral O1 and when the positive O1 is preceded by the neutral T1).

In line with earlier findings (e.g. [10,32]), our results are difficult to reconcile with associative models such as the Rescorla–Wagner model ([33]; see also [34]), which allow associations to weaken when contingencies no longer hold.^11^ Such models assume that associations between stimulus representations are formed during acquisition and are then destroyed during extinction or counterconditioning. The fact that a variety of extinction-like tasks did not reduce the magnitude of IR effects can be explained by associative models only if it is assumed that the S1–O1 and T1–O1 associations are not weakened by the S1 and T1 presentations during the extinction phase. Alternative models argue that ‘extinction involves new learning rather than unlearning and can still leave the original … responding susceptible to renewal (return of conditioned responding after a context change), spontaneous recovery (after the passage of time), and reinstatement (return after re-exposure to the US)’ [29, p. 52]. Yet even models that allow for the formation of new (context-dependent) inhibitory associations rather than the weakening of (context-independent) excitatory associations (e.g. [35]) would predict an impact of extinction procedures on IR effects and would thus be incompatible with our findings. Whereas many of these theoretical conclusions are supported not only by our findings but also by previous studies showing a lack of extinction of EC, our findings again add a new dimension because they necessitate the assumption of a backward spreading of activation across associations. For instance, it forces any associative model that would invoke inhibitory associations to make assumptions about whether and when activation can spread backwards across those associations. From this perspective, it would be interesting to pit an ‘unlearning’ against a ‘new inhibitory learning’ account of our extinction and counterconditioning findings by replicating our initial design and then including a third stage that assesses for phenomena such as recovery, reinstatement and renewal (evidence for which would support the latter over the former account).

In the context of EC, it has been argued that unlike most other types of learned behaviour, learned preferences depend on associations that reflect the number of stimulus co-occurrences but not events in which stimuli occur separately (see [36,37]). This idea could also account for the lack of extinction in our studies, but only if it is assumed that activation can spread in a backwards manner across these associations. Although one cannot exclude these associative accounts of resistance to extinction, they are largely *post hoc* and require additional assumptions about when which type of behaviour will be mediated by which type of associative mechanism. In summary, together with previous demonstrations of resistance to extinction in the EC literature, our findings constrain associative models of learning in important ways.

#### 6.3.2. Propositional (mental) models

Our results also constrain propositional accounts of evaluative learning [38–40]. Whereas associations (e.g. happy–sad) merely convey the strength with which representations are linked, propositions specify how objects are related and have a truth value (e.g. happy is opposite to sad). It may be that an IR-based learning procedure gives rise to the formation of two propositions based on the person's direct experience (e.g. ‘the positive source leads to the outcome’, ‘neutral targets lead to that same outcome’) and that these propositions set the stage for the generation of a third ‘inferred’ proposition about the evaluative properties of the stimuli (i.e. ‘positive sources and neutral targets are related, therefore the neutral targets are also positive’). It is this inferred proposition that mediates the subsequent change in liking (for more, see [41]).

The results of experiments 1–4 suggest that the latter inferred proposition may be maintained even when the premises of the inference (i.e. the propositions about the intersecting contingencies) no longer hold. Note that just like associative accounts of resistance to extinction, this propositional account is also highly speculative and *post hoc*. It does not specify why the inferred propositions would hold when the premises no longer hold. When it comes to counterconditioning, it may be that in experiment 5 (where the valenced source was reversed), a series of further propositions were formed based on the individual's novel experiences (e.g. ‘there is now a new source related to the outcome’) which in turn led to the formation of a new evaluative inference (e.g. ‘the target is negative’). This latter inference may counteract the effects of the original propositions and mediate the reversed IR effect. By contrast, rearranging the contingencies, as in experiments 6 and 7, may lead to the formation of propositions that are ambivalent in nature (e.g. ‘the target is sometimes related with positive and at other times with negative sources/outcomes'). These ambivalent propositions may lead to neutral stimulus evaluations such as we obtained in our final two experiments. Future work could put this idea to the test by investigating if different evaluative change procedures (e.g. counterconditioning, extinction) set the stage for different types of propositions, and if so, whether these propositions are related to the persistence or change of evaluative learning effects. In any case, because IR involve multiple regularities, each of which can be changed in extinction and counterconditioning procedures, propositional accounts of (extinction and counterconditioning of) learning via IR require multiple propositions about (changes in) multiple regularities, thus heavily constraining any possible propositional account of these effects.

### 6.4. Practical implications

The ultimate goal when changing evaluations is to demonstrate that doing so leads to a corresponding change in behaviour. For instance, an advertisement sets out to increase consumer liking of a brand product with the hope that this change in liking will lead people to actually purchase the product itself. Therefore, it seems useful to identify learning pathways that produce changes in liking that persist across time and in the face of extinction. Our data suggest that this is true for evaluative learning via IR and OEC, where changes in liking were still detectable even when the intersection or contingencies was subsequently disrupted. If anything, IR and OEC effects persisted in the face of extinction procedures. Thus, if a consumer product acquires a positive valence via IR or OEC, people may continue to like that item even when they later encounter it by itself in the supermarket. Likewise, if one's product has acquired a positive valence via IR, it may be resistant to change as well. Future work could take this idea one step further and compare IR and OEC to other known evaluative learning pathways (e.g. ME, EC, AA) to determine which pathway influences evaluations and behaviour to the greatest extent.

On a related note, it remains to be seen whether changes in self-reported and automatic evaluations via extinction or counterconditioning correlate with changes in other classes of (real-world) behaviour. So far, research on IR has mostly focused on establishing or changing evaluations and intentions towards novel stimuli (experiments 1–7) or pre-existing stimuli. For instance, Mattavelli *et al*. [42] used the self-referencing task, an IR-based paradigm in which stimuli are related with the (generally positive) concept of self, to countercondition green vegetables in a population of participants who did not like green vegetables. This intervention led to more positive implicit attitudes towards green vegetables and to an increased intention to consume them in future. Nevertheless, it remains to be seen if IR-based procedures are also effective when it comes to actual behavioural change (e.g. increased green vegetable consumption).

### 6.5. Limitations and future directions

One limitation was the difficulty we observed in creating an extinction procedure which effectively undermined evaluations of the target stimulus (IR effects). It may be that the extinction procedure used in experiments 1–3 still retained some valenced elements (e.g. the responses emitted in the presence of the source stimuli) which may have hampered our efforts to extinguish target evaluations. Experiment 4 sought to control for this possibility by presenting stimuli without the need to emit responses—but even this task is not without its own issues (e.g. presenting stimuli in a non-contingent way might be perceived as being unrelated to the acquisition phase; see our previous point about contextual versus a-contextual learning). Another possibility would be to simply omit the valenced contingencies entirely and just expose participants to the target contingencies during extinction. Or to replace the valenced source with a neutral source (although this may come close to the counterconditioning procedure used in experiment 5). In either case, future work could seek to build and refine on our initial efforts here.

Another limitation was the presence of both a ‘visible intersection’ (e.g. common outcome) and a ‘hidden intersection’ (i.e. common response locations) connecting the contingencies in many studies. This latter type of intersection may have augmented the IR and OEC effects during the acquisition phase and undermined attempts to reduce them during extinction and counterconditioning. That said, when this hidden intersection was absent (experiment 4) or controlled for (experiment 7), we still failed to observe extinction or counterconditioning. Nevertheless, we recognize that this factor probably played a role in the findings reported here. Future work should, therefore, control for and examine this issue more systematically, seeking to establish and change IR effects.

## 7. Conclusion

We examined the robustness of evaluations established via IR and OEC. Although we could generate novel evaluations via both learning pathways, we could not easily extinguish or countercondition those evaluations using variants of commonly used procedures. This supports the idea that, once formed, IR effects may be difficult to eliminate. The current work represents the first time, to our knowledge, that these recently discovered learning pathways have been examined in this way. We encourage others to further explore promising strategies for altering what people like and dislike.

## Supplementary Material

Supplementary Materials

Reviewer comments

## Footnotes

1 The concept of a ‘regularity’ is simply a term denoting any state ‘in the environment…that entails more than the presence of a single stimulus or behavior at a single moment in time’ ([8, p. 634]; for more on this topic, see [9]).

2 Unlike EC, where there are only two (conditioned (CS) and unconditioned (US)) stimuli involved, the intersecting regularities procedures reported here involve three key stimuli: the valenced *source* stimulus, neutral *outcome* stimulus and the neutral *target* stimulus. We will adopt the latter terminology from this point onwards (also see [7]).

3 Learning via intersecting regularities does not refer to a single procedure but rather a class of procedures that each set out to (i) create multiple regularities that (ii) intersect with one another in terms of a common element (e.g. stimulus or response). The work reported in this paper focuses on just one such procedure.

4 Extinction procedures in the context of classical and operant conditioning not only remove the regularity that originally gave rise to the change in behaviour but also (typically) remove the valenced stimulus as well. Many of the extinction procedures used here removed the regularity but retained the valenced stimulus (experiments 1–3), although one experiment did remove both regularity and valenced stimulus (experiment 4).

5 The procedures described in experiments 1–7 are—strictly speaking—not extinction or counterconditioning tasks, given that extinction and counterconditioning typically refer to procedures used in the classical and/or operant conditioning literatures and not to situations involving intersections between regularities. Rather than open a conceptual debate surround the meaning of these two terms, we were simply interested in testing the *robustness* of IR effects in the face of manipulations that attempt to undo (which is often the goal of extinction tasks) or modify (which is often the goal in counterconditioning tasks) the intersections that gave rise to the original IR effects. We will continue to refer to extinction- and counterconditioning-like tasks for communication sake.

6 Different types of OEC can be distinguished depending on what is the valenced event and what is the initially neutral event that acquires a new valence within a single operant contingency. In the present set of experiments, the valenced event is a stimulus that signals the nature of the correct response (i.e. the source) and the neutral event is the outcome of the response. In other types of OEC such as AA learning, the valenced event is the response (i.e., approaching or avoiding), whereas the neutral event is the stimulus that signals the correct response. In still other types of OEC, the outcome is the valenced event and the response or the stimulus signalling the response are the initially neutral event.

7 Note that the study designs and data analysis plans for all experiments are available on the Open Science Framework website (osf.io/u6vtz). We report all manipulations and measures used in our experiments. All data were collected without intermittent data analysis. The data analytic plan, experimental scripts and data are available at the above link. Deviations from pre-registration can also be found at the above link.

8 This procedure should extinguish IR effects but leave OEC effects intact. Indeed, if anything, the procedure may further strengthen OEC effects, given that it provides double the exposure to the contingencies underpinning OEC effects relative to the acquisition-only group.

9 Our original pre-registered plan was to simply assess for IR and OEC effects. However, a reviewer asked that we document how participants performed during the training and intervention phases, and show that they were also attentive throughout the entire learning task. We, therefore, assessed for the mean accuracy within each phase (table 1) and calculated a ‘pass criterion’ (at least 75% on the final block of a given phase; see table 2). Although this latter criterion is *post hoc* and others could certainly be chosen, we believe that it provides a useful means of distinguishing between those who discriminated the stimulus–response and response–outcomes relations versus those who did not (a similar criterion was used by Hughes *et al*. [6]).

10 The counterconditioning procedure in experiment 6 should impact outcome and target stimuli in different ways. It could potentially reverse evaluations of target stimuli while leaving intact (or strengthening) previously acquired outcome evaluations (i.e. countercondition IR effects while boosting OEC effects, given that it involves additional exposure to the same operant evaluative conditioning contingencies as in the acquisition phase).

11 Note that in these models, it is not only the regularity that originally gave rise to the change in behaviour that is removed but also (typically) the valenced stimulus as well (whereas in our case the valenced stimulus was often still present).

## Data accessibility

Our data, materials and code can be found at osf.io/u6vtz.
