# Supplementary material for: The influence of extinction and counterconditioning procedures on operant evaluative conditioning and intersecting regularity effects
Source: R Soc Open Sci. 2020 Oct 7;7(10):192085. doi: 10.1098/rsos.192085 (PMC7657930; doi:10.1098/rsos.192085)

**The Influence of Extinction and Counterconditioning Procedures on Operant Evaluative Conditioning and Intersecting Regularity Effects**

**Hughes, Mattavelli, Hussey, & De Houwer (2020)**

***Supplementary Materials***

**Sensitivity Meta-Analyses**

In these supplementary analyses we first excluded participants who failed to meet mastery criteria in the final block of the training or testing phases (>75% accuracy). We then conducted a similar set of analyzes as reported in the main manuscript, but this time exclusively on the data of those who passed the above criterion (i.e., on the ‘pass’ group; for our rational in doing so see the ‘meta-analysis’ section of the main manuscript). Thereafter we compared the results obtained from this ‘pass group’ to the overall sample reported in the main manuscript, in order to determine if the conclusions we arrived at in the latter differ from those obtained with the former. This question was only considered at the meta-analytic level as sample sizes were likely under-powered to answer it at the individual study level.

*Table 1*. Overview of the number of participants who were excluded from the sensitivity analyses because they failed to achieve the pass criterion on the final block of the acquisition training or testing phases.

| Experiment | Participants with complete data | Participants subsequently excluded because they failed training or testing | % Excluded |
| --- | --- | --- | --- |
| 1 | 97 | 22 | 22.7 |
| 2 | 94 | 15 | 16.0 |
| 3 | 95 | 23 | 24.2 |
| 4 | 98 | 20 | 20.4 |
| 5 | 95 | 17 | 17.9 |
| 6 | 90 | 12 | 13.3 |
| 7 | 313 | 92 | 29.4 |
| Total | 882 | 201 | 22.8 |

## **Question 1: Do the Conclusions We Make about the Presence of OEC and IR Effects *in General* Differ Between the Total Sample Relative to the Pass Group?**

## In our original analyses we wanted to know if operant evaluative conditioning and intersecting regularities gave rise to novel evaluations *in general* (i.e., regardless of the specific measure used). In the supplementary analyses we wanted to know if the conclusions we originally made on the basis of the total sample differ when we only consider the pass group. We first re-ran the same set of analyses as reported in the main manuscript, but this time only on the pass group’s OEC and IR effects. Once again OEC and IR effects consistently emerged across studies (see Figure 1). We then compared the effects obtained in the pass group to those obtained in the total sample to see if they differed from one another. Conclusions about the significance of the estimate of the effect were congruent between the meta-analysis of the total sample and the sensitivity analysis run on the pass group.

**Figure 1**

*Meta-analytic models outlining the IR and OEC effects for those in the pass group. In each forest plot, squares represent observed Cohen’s d effect sizes, size of square represents weighting in the model, and error bars represent 95% Confidence Intervals (CIs) around the effect size.*


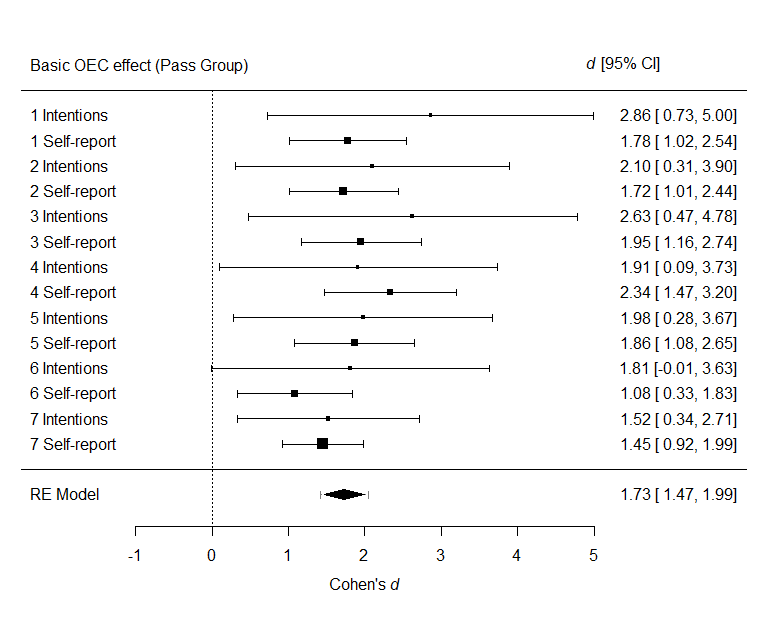

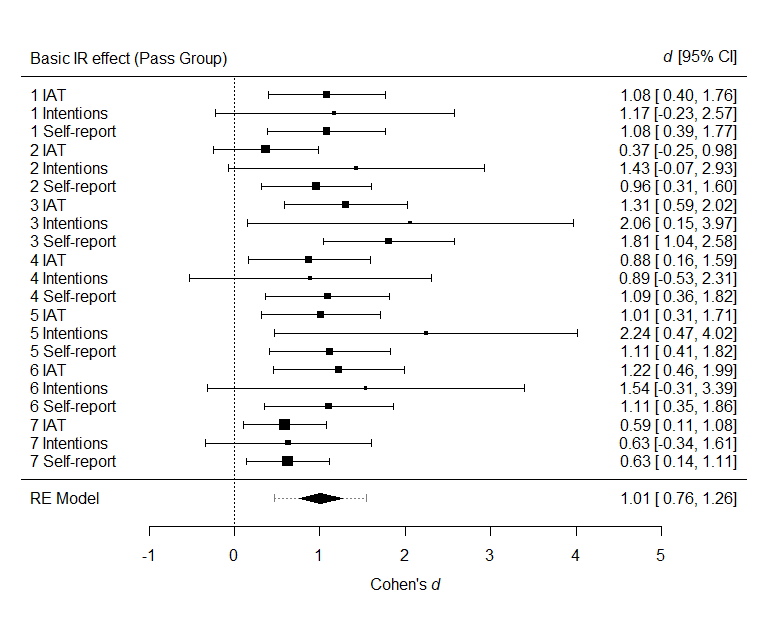


## **Question 2: Do the Conclusions we Made about OEC and IR Effects Being Moderated by Extinction or Counterconditioning Differ for the Total Sample Relative to the Pass Group?**

In our original analysis we wanted to know if the extinction and counterconditioning procedures we used moderate evaluations established via intersecting regularities *in general*? In the supplementary analyses we wanted to know if the conclusions we originally made on the basis of the total sample differ when we only consider the pass group. We first re-ran the same set of analyses as reported in the main manuscript, but this time only on the pass group’s data (see Figure 2).

***Extinction***

The meta-analytic model indicated that, in general, there was no evidence to support the idea that OEC effects, *d* = 0.05, 95% CI [-0.15, 0.24], *p* = .63, nor IR effects, *d* = .04 , 95% CI [-0.18, 0.26], *p* = .72, were moderated by the extinction procedures used in this paper.

***Counterconditioning***

The meta-analytic model indicated that, in general, there was no strong evidence to support the idea that IR effects were moderated by the counterconditioning procedures used in this paper, *d* = -0.17, 95% CI [-0.35, 0.01], *p* = .06.

**Sensitivity Analyses**

We then compared the effects obtained in the pass group to those obtained in the total sample to see if they differed from one another. Conclusions about the significance of the estimate of the effect were congruent between the meta-analysis of the total sample and the sensitivity analysis run on the pass group.

**Figure 2**

*Meta-analytic models outlining moderation of the IR and OEC effects by intervention type (extinction [top panels] or counterconditioning [bottom panels]) for the pass group. In each forest plot, squares represent observed Cohen’s d effect sizes, size of square represents weighting in the model, and error bars represent 95% Confidence Intervals (CIs) around the effect size.*


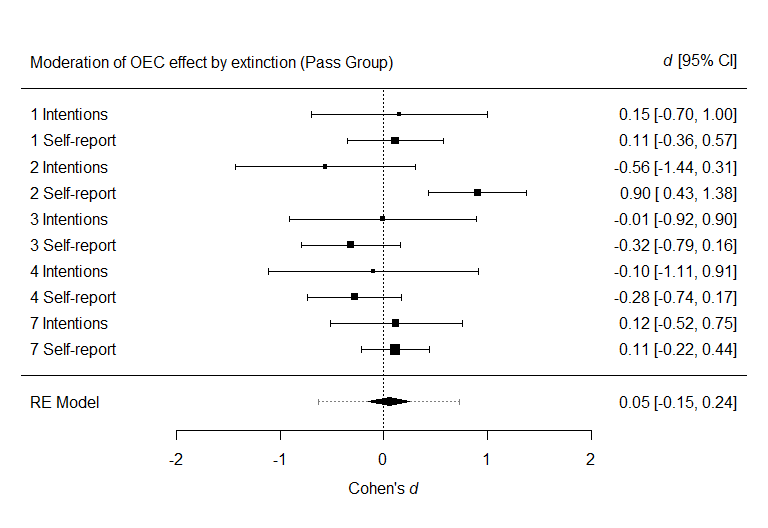

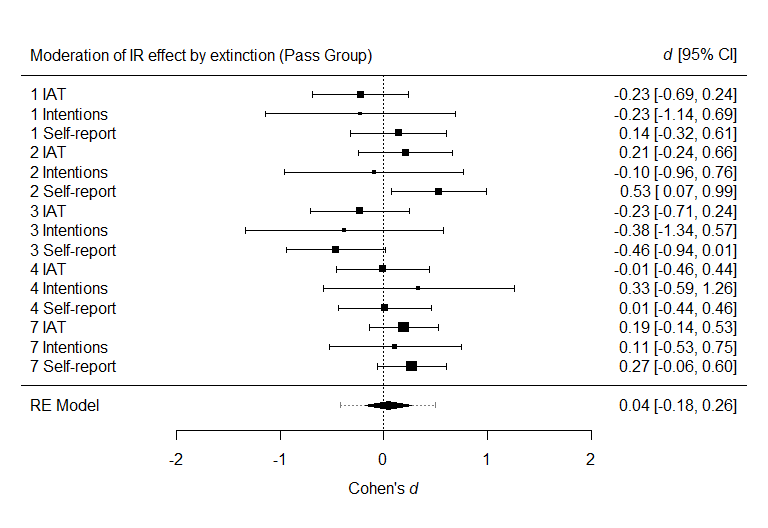

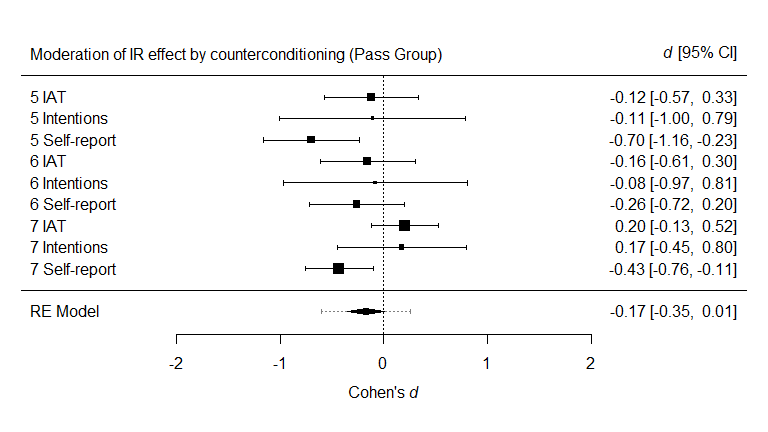

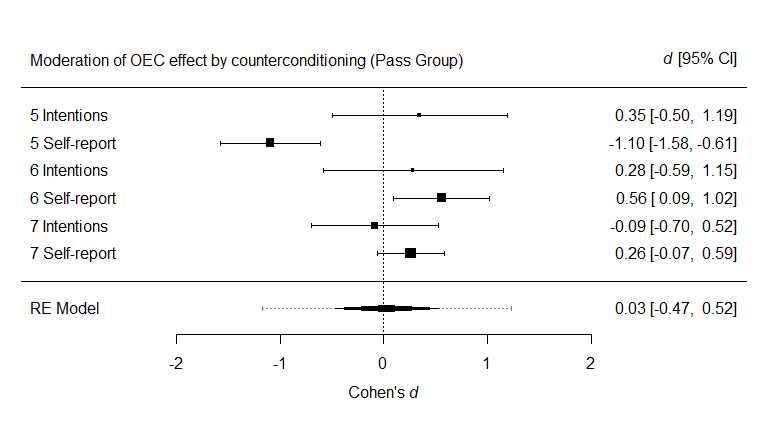

Supplement: Supplementary Materials [file rsos192085supp1.docx]
