# Supplementary material for: The influence of extinction and counterconditioning procedures on operant evaluative conditioning and intersecting regularity effects
Source: R Soc Open Sci. 2020 Oct 7;7(10):192085. doi: 10.1098/rsos.192085 (PMC7657930; doi:10.1098/rsos.192085)
Supplement: Reviewer comments [file rsos192085_review_history.pdf]

THE ROYAL SOCIETY
PUBLISHING

ROYAL SOCIETY
OPEN SCIENCE

The influence of extinction and counterconditioning procedures on operant evaluative conditioning and intersecting regularity effects

Sean Hughes, Simone Mattavelli, Ian Hussey and Jan De Houwer

Article citation details
R. Soc. open sci. **7**: 192085.
http://dx.doi.org/10.1098/rsos.192085

Review timeline
Original submission: 29 November 2019
Revised submission: 1 September 2020
Final acceptance: 9 September 2020

Note: Reports are unedited and appear as submitted by the referee. The review history appears in chronological order.

Review History

RSOS-192085.R0 (Original submission)

Review form: Reviewer 1

Is the manuscript scientifically sound in its present form?
Yes

Are the interpretations and conclusions justified by the results?
Yes

Is the language acceptable?
Yes

Do you have any ethical concerns with this paper?
No

Have you any concerns about statistical analyses in this paper?
No

Recommendation?
Accept as is

Reports © 2020 The Reviewers; Decision Letters © 2020 The Reviewers and Editors; Responses © 2020 The Reviewers, Editors and Authors. Published by the Royal Society under the terms of the Creative Commons Attribution License http://creativecommons.org/licenses/by/4.0/, which permits unrestricted use, provided the original author and source are credited

Comments to the Author(s)
I found all of my concerns adequatly adressed by the authors and belive that this makes a fine paper. Due to their clarifications, I now find the intersecting regularities perspective quite inspiring.

Review form: Reviewer 2 (Anand Krishna)

Is the manuscript scientifically sound in its present form?
Yes

Are the interpretations and conclusions justified by the results?
Yes

Is the language acceptable?
Yes

Do you have any ethical concerns with this paper?
No

Have you any concerns about statistical analyses in this paper?
Yes

Recommendation?
Accept with minor revision (please list in comments)

Comments to the Author(s)
In their manuscript "The Influence of Extinction and Counterconditioning on Operant Evaluative Conditioning and Intersecting Regularity Effects", Hughes and colleagues discuss a large set of experiments in which evaluation changes are acquired via intersecting regularities. These changes are then subjected to various systematically varied interventions designed to reverse them in order to test determinants of their robustness.
Coming in as a new reviewer on a revision is always a challenge. I have studied the comments by the previous reviewers and compared them to the manuscript's current form. In my view, the authors have successfully addressed the issues raised in the previous reviews. For my own part, I find the paper examines a scientifically relevant question with a strong empirical basis. While this kind of nitty-gritty research isn't always the most glamourous, it is important to carefully dissect the operating circumstances of these processes.
Therefore, I only have one concern before I can recommend this manuscript for acceptance, namely that I don't understand how the authors get 47 degrees of freedom from a 51 person sample in their pretest (p.18 of the pdf). This is likely a simple error to correct.
Otherwise, a few typos:
p.37 "beyond the constrains" should be "beyond the constraints"
p.39 "account for the lack of extinction in our studies be it that forces them" – this makes no grammatical sense as written
Finally, the sheer amount of analyses over so many studies and dependent variables can be quite tiring as a reader – as a quality-of-life improvement, I think summarizing non-significant results in the main text in a simple sentence and presenting the full analyses in an appendix might be useful.

Beyond these few issues, I recommend the manuscript for publication.
Signed
Anand Krishna

Review form: Reviewer 3

Is the manuscript scientifically sound in its present form?
Yes

Are the interpretations and conclusions justified by the results?
Yes

Is the language acceptable?
Yes

Do you have any ethical concerns with this paper?
No

Have you any concerns about statistical analyses in this paper?
Yes

Recommendation?
Major revision is needed (please make suggestions in comments)

Comments to the Author(s)
This study has widely examined the capacity of several variants of (analogues to) extinction and counterconditioning to diminish or reverse the evaluation formed via intersecting regularities. The paper is mostly clear and easy to read. I praise the authors for placing all the materials related to the study in an organized and comprehensive manner, which is helps to promote open science.
It is evident that a considerable amount of work has been invested in this work. Multiple experiments were designed and executed to tackle the research question. This resulted in a relatively comprehensive work that helps to extend our knowledge about associative learning and in particular about the malleability of conditioned evaluations formed through evaluative (operant) conditioning and an extension of it termed intersecting regularities. Notably, the authors perform meta-analyses on the results of the seven experiments conducted in this study which further inform the reader.
Nevertheless, this work includes a few methodological problems which should be taken in consideration and a few clarifications are needed with regard to several parts of this work.
I raise here a few main issues and some minor issues/corrections/clarifications needed as well as a few suggestions to potentially better communicate this work.
I start with a few general notes and then my comments mostly follow to different parts of the manuscript (where most but not all comments are minor).

General comments:
- A substantial methodological problem in this study is the additional intersecting component of the side right or left (accompanied with the use of the right or left hand). This was recognized by the authors who committed an attempt to address it in experiment 7, which if anything, demonstrated the importance of this factor. This intersection was presented along experiments 1 to 6 during training and in experiments 1-3 and 6 also during extinction/counterconditioning (* but notably not in experiment 5, unlike what the authors indicated in p. 29). This is a serious confound that compel us to take the conclusions drawn from this work with a grain of salt and creates a special necessity to verify them in future work. In my view this point should be highlighted throughout the manuscript wherever appropriate (currently it is firstly mentioned in the description of experiment 7) and accordingly the conclusions should be described with caution and with a proportional amount of confidence. On a side none, with regard to experiment 3, where extinction was effective, the presence of an additional intersection possibly suggests that the type of extinction procedure used in this case has high efficacy.

- Since the experiments were preformed on-line, special measures are required to ensure participants' engagement. The only exclusion criteria with regard to participants engagement were based on the IAT (except for experiment 7). Learning criteria for the acquisition and extinction/counterconditioning phases are required, or at least providing evidence that all the participants were engaged during these phases. Specifying mean and SD for accuracy and indicate whether there are outliers as well as testing that removing those outliers does not affect the results would be satisfying. Also, as this could not be done for experiment 4 (passive viewing of the neutral stimuli), this should be discussed as a potential cause for the lack of extinction.
- Throughout the paper in general and the discussion in particular the results of experiment 3 are largely ignored.
- Please make the pre-registration documents accessible as currently accessing them requires permission.
- I tried to reproduce the results to gain a better understanding of some analyses but was unable to because there are only data files (or sav files with no log). I highly suggest to provide an easy way to reproduce your results (you can add the sass script you have used for instance).

Introduction
- Some more background information about operant evaluative conditioning will be useful.
- Although adopted from a previous study, the operant conditioning procedure used here is not trivial. Traditionally in operant conditioning a subject is exposed to a neutral stimulus and a response is chosen freely and is followed by a valenced stimulus and not vice vera. I think that a few words about why choosing this procedure and what role does the response play here would help orienting the reader.
- p. 5 With regard to counterconditioning "During a first phase (acquisition) a contingency is established between two stimuli by pairing a neutral CS with a positive US. " Note that for clarity you should mention the opposite option of a negative US as it is referred in the subsequent lines.
- p.5 "This work was not designed to differentiate between two competing cognitive theories" - it is not clear in this context what are the competing cognitive theories are you referring to.
- The examples for extinction and counterconditioning are given in the form of classical conditioning whereas you this study is using operant conditioning. Therefore, I recommend to set these examples in the context of operant conditioning.
- p.6 "We refer to this as an extinction-like procedure because, similar to extinction tasks in EC, it involves the removal of the environmental event that underlies the target evaluation (in this case the common element shared by regularities)". This is true, yet notably, extinction traditionally removes the valenced stimulus. I propose to indicate this important distinction.
- The IAT is not explained clearly enough (e..g, in the first two blocks there is only one item on each side and is not clear from text). Please edit accordingly and/or add a figure with illustration.
- p. 7 "We added the IAT because it is assumed to capture more automatic instances of evaluation that can influence behavior in unique ways" unique ways is rather vague, I suggest to elaborate (or rephrase).

Methods
- For each experiment please report how many participants were assigned to each group.
- Please report the power analysis / N justification in the main text or at least mention it and refer to experiment's repository. Also, please specify when data collection was stopped for each sample (was there a guiding rule).
- Is there a reason for the discrepancy in the error feedback duration between extinction and acquisition (3s vs 2s)?
- p. 11 With regard to the behavioral intention task, it is not entirely clear which items/stimuli were compared and in which manner. Please describe the task more comprehensively (a figure illustrating the task is also an option).
- p. 12 Exploratory questions section - although exploratory, as the memory test was also conducted between the acquisition and the following extinction/counterconditioning phase, one may be concerned that it may affect the evaluation results. Therefore, please describe the test. Additionally, were the final memory test and the other exploratory measures conducted after recording the evaluative measures?

- Experiment. 5 p. 24 - "In all cases these were similar to those reported in Experiments 1-5 unless otherwise stated. " I believe this should be Experiments 1-4.
- For experiment 7, why the procedure chosen for the counterconditioning was adopted from experiment 6 rather than experiment 5 which yielded more promising results?

Analysis
- In p. 7 the authors state "A 2 (Stimulus: neutral target related to positive vs. negative source) x 2 (Training: Extinction vs. Acquisition-only) mixed design was employed with the first factor measured within and the second measured between participants." Yet later, when reporting the Self-report ratings they state they indicate using "A one way ANOVA on target stimulus ratings (T1 and T2) with Training Condition as a between participants factor" - is it actually a mixed design, or is the DV is the difference between T1 and T2 (in that case there is no point in running ANOVA rather than a t-test on the two groups; this is also relevant for the IAT analysis). Please clarify this point.
- With regard to the logistic regression analysis, it would helpful if the model would be specified. Also, to determine whether there is a main effect of training a model comparison should be employed (between a model with and model without this factor).
- The meta analysis for the IR is highly informing. However, for the EOC the meta analysis is flawed as in some experiments extinction and counterconditioning were simply additional training sessions for the EOC contingencies.

Results
- Experiment 3, p.20 - "Intentions emerged in the expected direction in both the acquisition-only… and extinction conditions". This is somewhat misleading as the expected direction in this experiment for the OEC following extinction is different from following the acquisition.
- Experiment 4. p. 21 - "Each trial began with the presentation of a stimulus (T1, O1, T2, O2) for 1500ms (each stimulus was presented five times per block). " The word began is a bit misleading here as each trial includes only the presentation of a single stimulus.
- Experiment 7 - please indicate how many participants required a fifth block and what was the overall success rate.
- Experiment 7 - It is not entirely clear whether on extinction and counterconditioning the addition of a fifth block was dependent on the same condition as in training (please indicate explicitly in the text).
- Experiment 7 - please indicate when throughout the experiment you integrated the matching to sample (MTS) task.
- Experiment 7, p. 31 under Self-report ratings (intersecting regularities) - it seems that the degrees of freedom in the F-test are incorrect. Please check also the other reports of degrees of freedom in the results of this experiment as I suspect there other mistakes.

General Discussion:
- With regard to the theoretical discussion about the implications of this study, have the authors considered the option that the outcome becomes a secondary reinforcer upon acquiring the positive/negative value and thereby reinforce the target (neutral) stimulus? Would such case be reconciled with the potential theoretical implications discussed in the text or would use as an alternative explanation?
- The issue of the additional left/right side (and hand) intersection must be referred to in the general discussion, especially when referring to the ineffectiveness of the extinction.
- p. 35 - "evaluations… were reduced by counterconditioning-like procedures." In practice only one procedure yield a clear counterconditiong effect.
- p. 37 - "In line with earlier findings (e.g., Pavlov, 1927; Baeyens et al., 1988), our results are difficult to reconcile with associative models such as the Rescorla-Wagner model (Rescorla & Wagner, 1972; see also McCloskey & Cohen, 1989), which allow associations to weaken when contingencies no longer hold." When stating this I think the authors should make the distinction that in the models mentioned the valenced part (the US) is the one traditionally removed from the contingency.

- p.38 - "The fact that a variety of extinction-like tasks did not reduce the magnitude of IR effects (Experiments 1, 2, and 4) can be explained by associative models only if it is assumed that the S1-O1 and T1-O1 associations are not weakened by the S1 and T1 presentations during the extinction phase." But is it not the case that the very same explanation is contrasted in experiment 3?
- pp. 38-39 - "Resistance to extinction could also be accounted for on the basis of comparator-type models (e.g., Miller & Matzel, 1988) if one assumes that, unlike most other types of learned behavior, learned preferences do not depend on the output of a comparator mechanism, but directly reflect the strength of individual associations (e.g., De Houwer, 1998). " I find the notion about comparator models here somewhat self-contradictory or unclear, please explain/elaborate.
- p.39 - "The results of Experiments 1-4 suggest that the latter inferred proposition may be maintained even when the premises of the inference (i.e., the propositions about the intersecting contingencies) no longer hold. " It seems to me that this statement does not hold for experiment 3.
- A theoretical account for (or an attempt to explain) the success of extinction in experiment 3 compared to 1 and 2 could be insightful. Same goes for the success of counterconditioning in experiment 5 compared to 6 and 7.
- p. 43 "It would be worth examining the long-term effects and context dependency of counterconditioning via IR in order to determine if these changes in evaluation are stable across time and context." Note that this was already suggested earlier in the discussion. For clarity you can just add "as mentioned before" or something of that sort.
- p. 44. "We also consistently manipulated parameters of the extinction and counterconditioning tasks from one study to the next which (a) may have made the task increasingly taxing on participants ". Can you please elaborate on that?

Conclusion (p. 45):
- I think that in light of the variety of methods used and results obtained that the conclusion should be conveyed in a less deterministic manner with regard to the potential ability of exitinction and counterconditioning to change the evaluations formed through IR.
- The authors indicate that the "Same evaluations can be counterconditioned by… or (to a lesser extent) by reconfiguring the intersection". However, when tested separately "reconfiguring the intersection" did not significantly change evaluations so this may draw confusion.

Suggestions for communicating purposes and to increase clarity:
- I suggest to include a figure illustrating the procedure/s, as common in papers reporting human behavioral experiments.
- As this experiment involves multiple experiments with multiple dependent variables for each experiment I would highly recommend to put together all the main results in some kind of a visualized table or plot indicating where significant results were found. One or a few barplots summarizing the results are also an option.
- The exclusions throughout the experiments are based on the same criteria, thus it is possible to add a table of exclusions by criterion for all the experiments and shorten the recurrent detailed description of them throughout the text.
- Keep using the specific keyboard key names as constantly associated with the same positive/negative stimuli throughout the text gives the wrong impression that this factor not counterbalanced (although it is stated that it was).

Please add relevant citations in the following cases:
- When mentioning preconditioning in the introduction.
- In p.35 for this statement: "This finding parallels result in evaluative conditioning studies in which the impact of counterconditioning procedures has also proven to be more robust than that of extinction procedures."
- In p.38 for this statement: "Whereas many of these theoretical conclusions are supported not only by our findings but also by previous studies showing a lack of extinction of evaluative conditioning".

Decision letter (RSOS-192085.R0)

26-Feb-2020

Dear Dr Hughes

On behalf of the Editors, I am pleased to inform you that your Manuscript RSOS-192085 entitled "The Influence of Extinction and Counterconditioning on Operant Evaluative Conditioning and Intersecting Regularity Effects" has been accepted for publication in Royal Society Open Science subject to minor revision in accordance with the referee suggestions. Please find the referees' comments at the end of this email.

The reviewers and handling editors have recommended publication, but also suggest some minor revisions to your manuscript. Therefore, I invite you to respond to the comments and revise your manuscript.

• Ethics statement
If your study uses humans or animals please include details of the ethical approval received, including the name of the committee that granted approval. For human studies please also detail whether informed consent was obtained. For field studies on animals please include details of all permissions, licences and/or approvals granted to carry out the fieldwork.

• Data accessibility
It is a condition of publication that all supporting data are made available either as supplementary information or preferably in a suitable permanent repository. The data accessibility section should state where the article's supporting data can be accessed. This section should also include details, where possible of where to access other relevant research materials such as statistical tools, protocols, software etc can be accessed. If the data has been deposited in an external repository this section should list the database, accession number and link to the DOI for all data from the article that has been made publicly available. Data sets that have been deposited in an external repository and have a DOI should also be appropriately cited in the manuscript and included in the reference list.

If you wish to submit your supporting data or code to Dryad (http://datadryad.org/), or modify your current submission to dryad, please use the following link:
http://datadryad.org/submit?journalID=RSOS&manu=RSOS-192085

• Competing interests
Please declare any financial or non-financial competing interests, or state that you have no competing interests.

• Authors' contributions
All submissions, other than those with a single author, must include an Authors' Contributions section which individually lists the specific contribution of each author. The list of Authors should meet all of the following criteria; 1) substantial contributions to conception and design, or acquisition of data, or analysis and interpretation of data; 2) drafting the article or revising it critically for important intellectual content; and 3) final approval of the version to be published.

All contributors who do not meet all of these criteria should be included in the acknowledgements.

We suggest the following format:
AB carried out the molecular lab work, participated in data analysis, carried out sequence alignments, participated in the design of the study and drafted the manuscript; CD carried out the statistical analyses; EF collected field data; GH conceived of the study, designed the study,

coordinated the study and helped draft the manuscript. All authors gave final approval for publication.

• Acknowledgements
Please acknowledge anyone who contributed to the study but did not meet the authorship criteria.

• Funding statement
Please list the source of funding for each author.

Please ensure you have prepared your revision in accordance with the guidance at https://royalsociety.org/journals/authors/author-guidelines/ -- please note that we cannot publish your manuscript without the end statements. We have included a screenshot example of the end statements for reference. If you feel that a given heading is not relevant to your paper, please nevertheless include the heading and explicitly state that it is not relevant to your work.

Because the schedule for publication is very tight, it is a condition of publication that you submit the revised version of your manuscript before 06-Mar-2020. Please note that the revision deadline will expire at 00.00am on this date. If you do not think you will be able to meet this date please let me know immediately.

To revise your manuscript, log into https://mc.manuscriptcentral.com/rsos and enter your Author Centre, where you will find your manuscript title listed under "Manuscripts with Decisions". Under "Actions," click on "Create a Revision." You will be unable to make your revisions on the originally submitted version of the manuscript. Instead, revise your manuscript and upload a new version through your Author Centre.

When submitting your revised manuscript, you will be able to respond to the comments made by the referees and upload a file "Response to Referees" in "Section 6 - File Upload". You can use this to document any changes you make to the original manuscript. In order to expedite the processing of the revised manuscript, please be as specific as possible in your response to the referees. We strongly recommend uploading two versions of your revised manuscript:

1) Identifying all the changes that have been made (for instance, in coloured highlight, in bold text, or tracked changes);
2) A 'clean' version of the new manuscript that incorporates the changes made, but does not highlight them.

When uploading your revised files please make sure that you have:

1) A text file of the manuscript (tex, txt, rtf, docx or doc), references, tables (including captions) and figure captions. Do not upload a PDF as your "Main Document";
2) A separate electronic file of each figure (EPS or print-quality PDF preferred (either format should be produced directly from original creation package), or original software format);
3) Included a 100 word media summary of your paper when requested at submission. Please ensure you have entered correct contact details (email, institution and telephone) in your user account;
4) Included the raw data to support the claims made in your paper. You can either include your data as electronic supplementary material or upload to a repository and include the relevant doi within your manuscript. Make sure it is clear in your data accessibility statement how the data can be accessed;
5) All supplementary materials accompanying an accepted article will be treated as in their final form. Note that the Royal Society will neither edit nor typeset supplementary material and it will be hosted as provided. Please ensure that the supplementary material includes the paper details where possible (authors, article title, journal name).

Supplementary files will be published alongside the paper on the journal website and posted on the online figshare repository (https://rs.figshare.com/). The heading and legend provided for each supplementary file during the submission process will be used to create the figshare page, so please ensure these are accurate and informative so that your files can be found in searches. Files on figshare will be made available approximately one week before the accompanying article so that the supplementary material can be attributed a unique DOI.

Please note that Royal Society Open Science charge article processing charges for all new submissions that are accepted for publication. Charges will also apply to papers transferred to Royal Society Open Science from other Royal Society Publishing journals, as well as papers submitted as part of our collaboration with the Royal Society of Chemistry (https://royalsocietypublishing.org/rsos/chemistry).

If your manuscript is newly submitted and subsequently accepted for publication, you will be asked to pay the article processing charge, unless you request a waiver and this is approved by Royal Society Publishing. You can find out more about the charges at https://royalsocietypublishing.org/rsos/charges. Should you have any queries, please contact openscience@royalsociety.org.

Once again, thank you for submitting your manuscript to Royal Society Open Science and I look forward to receiving your revision. If you have any questions at all, please do not hesitate to get in touch.

Kind regards,
Anita Kristiansen
Editorial Coordinator

Royal Society Open Science
openscience@royalsociety.org

on behalf of Dr Joydeep Bhattacharya (Associate Editor) and Essi Viding (Subject Editor)
openscience@royalsociety.org

Associate Editor Comments to Author (Dr Joydeep Bhattacharya):
Comments to the Author:
All reviewers found merits in your revised version, and especially, the experimental rigor has been highlighted. The reviewers and I appreciate the extensive amount of work you've put in this submitted work. However, there are still some important issues (especially a potential confound as mentioned by the Reviewer-3) that need to be clarified; further, Reviewer-3 has also made some useful suggestions for your considerations for improving the clarity. I am looking forward to receiving a revised final version.

Reviewer comments to Author:
Reviewer: 1
Comments to the Author(s)
I found all of my concerns adequatly adressed by the authors and belive that this makes a fine paper. Due to their clarifications, I now find the intersecting regularities perspective quite inspiring.

Reviewer: 2
Comments to the Author(s)
In their manuscript “The Influence of Extinction and Counterconditioning on Operant Evaluative Conditioning and Intersecting Regularity Effects”, Hughes and colleagues discuss a large set of experiments in which evaluation changes are acquired via intersecting regularities. These

changes are then subjected to various systematically varied interventions designed to reverse them in order to test determinants of their robustness.
Coming in as a new reviewer on a revision is always a challenge. I have studied the comments by the previous reviewers and compared them to the manuscript's current form. In my view, the authors have successfully addressed the issues raised in the previous reviews. For my own part, I find the paper examines a scientifically relevant question with a strong empirical basis. While this kind of nitty-gritty research isn't always the most glamourous, it is important to carefully dissect the operating circumstances of these processes.
Therefore, I only have one concern before I can recommend this manuscript for acceptance, namely that I don't understand how the authors get 47 degrees of freedom from a 51 person sample in their pretest (p.18 of the pdf). This is likely a simple error to correct.
Otherwise, a few typos:
p.37 "beyond the constrains" should be "beyond the constraints"
p.39 "account for the lack of extinction in our studies be it that forces them" – this makes no grammatical sense as written
Finally, the sheer amount of analyses over so many studies and dependent variables can be quite tiring as a reader – as a quality-of-life improvement, I think summarizing non-significant results in the main text in a simple sentence and presenting the full analyses in an appendix might be useful.

Beyond these few issues, I recommend the manuscript for publication.
Signed
Anand Krishna

Reviewer: 3
Comments to the Author(s)
This study has widely examined the capacity of several variants of (analogues to) extinction and counterconditioning to diminish or reverse the evaluation formed via intersecting regularities. The paper is mostly clear and easy to read. I praise the authors for placing all the materials related to the study in an organized and comprehensive manner, which is helps to promote open science.
It is evident that a considerable amount of work has been invested in this work. Multiple experiments were designed and executed to tackle the research question. This resulted in a relatively comprehensive work that helps to extend our knowledge about associative learning and in particular about the malleability of conditioned evaluations formed through evaluative (operant) conditioning and an extension of it termed intersecting regularities. Notably, the authors perform meta-analyses on the results of the seven experiments conducted in this study which further inform the reader.
Nevertheless, this work includes a few methodological problems which should be taken in consideration and a few clarifications are needed with regard to several parts of this work.
I raise here a few main issues and some minor issues/corrections/clarifications needed as well as a few suggestions to potentially better communicate this work.
I start with a few general notes and then my comments mostly follow to different parts of the manuscript (where most but not all comments are minor).

General comments:
- A substantial methodological problem in this study is the additional intersecting component of the side right or left (accompanied with the use of the right or left hand). This was recognized by the authors who committed an attempt to address it in experiment 7, which if anything, demonstrated the importance of this factor. This intersection was presented along experiments 1 to 6 during training and in experiments 1-3 and 6 also during extinction/counterconditioning (* but notably not in experiment 5, unlike what the authors indicated in p. 29). This is a serious confound that compel us to take the conclusions drawn from this work with a grain of salt and creates a special necessity to verify them in future work. In my view this point should be highlighted throughout the manuscript wherever appropriate (currently it is firstly mentioned in the description of experiment 7) and accordingly the conclusions should be described with

caution and with a proportional amount of confidence. On a side none, with regard to experiment 3, where extinction was effective, the presence of an additional intersection possibly suggests that the type of extinction procedure used in this case has high efficacy.
- Since the experiments were preformed on-line, special measures are required to ensure participants' engagement. The only exclusion criteria with regard to participants engagement were based on the IAT (except for experiment 7). Learning criteria for the acquisition and extinction/counterconditioning phases are required, or at least providing evidence that all the participants were engaged during these phases. Specifying mean and SD for accuracy and indicate whether there are outliers as well as testing that removing those outliers does not affect the results would be satisfying. Also, as this could not be done for experiment 4 (passive viewing of the neutral stimuli), this should be discussed as a potential cause for the lack of extinction.
- Throughout the paper in general and the discussion in particular the results of experiment 3 are largely ignored.
- Please make the pre-registration documents accessible as currently accessing them requires permission.
- I tried to reproduce the results to gain a better understanding of some analyses but was unable to because there are only data files (or sav files with no log). I highly suggest to provide an easy way to reproduce your results (you can add the sass script you have used for instance).

Introduction
- Some more background information about operant evaluative conditioning will be useful.
- Although adopted from a previous study, the operant conditioning procedure used here is not trivial. Traditionally in operant conditioning a subject is exposed to a neutral stimulus and a response is chosen freely and is followed by a valenced stimulus and not vice vera. I think that a few words about why choosing this procedure and what role does the response play here would help orienting the reader.
- p. 5 With regard to counterconditioning "During a first phase (acquisition) a contingency is established between two stimuli by pairing a neutral CS with a positive US. " Note that for clarity you should mention the opposite option of a negative US as it is referred in the subsequent lines.
- p.5 "This work was not designed to differentiate between two competing cognitive theories" - it is not clear in this context what are the competing cognitive theories are you referring to.
- The examples for extinction and counterconditioning are given in the form of classical conditioning whereas you this study is using operant conditioning. Therefore, I recommend to set these examples in the context of operant conditioning.
- p.6 "We refer to this as an extinction-like procedure because, similar to extinction tasks in EC, it involves the removal of the environmental event that underlies the target evaluation (in this case the common element shared by regularities)". This is true, yet notably, extinction traditionally removes the valenced stimulus. I propose to indicate this important distinction.
- The IAT is not explained clearly enough (e..g, in the first two blocks there is only one item on each side and is not clear from text). Please edit accordingly and/or add a figure with illustration.
- p. 7 "We added the IAT because it is assumed to capture more automatic instances of evaluation that can influence behavior in unique ways" unique ways is rather vague, I suggest to elaborate (or rephrase).

Methods
- For each experiment please report how many participants were assigned to each group.
- Please report the power analysis / N justification in the main text or at least mention it and refer to experiment's repository. Also, please specify when data collection was stopped for each sample (was there a guiding rule).
- Is there a reason for the discrepancy in the error feedback duration between extinction and acquisition (3s vs 2s)?
- p. 11 With regard to the behavioral intention task, it is not entirely clear which items/stimuli were compared and in which manner. Please describe the task more comprehensively (a figure illustrating the task is also an option).
- p. 12 Exploratory questions section - although exploratory, as the memory test was also conducted between the acquisition and the following extinction/counterconditioning phase, one

may be concerned that it may affect the evaluation results. Therefore, please describe the test. Additionally, were the final memory test and the other exploratory measures conducted after recording the evaluative measures?
- Experiment. 5 p. 24 - "In all cases these were similar to those reported in Experiments 1-5 unless otherwise stated. " I believe this should be Experiments 1-4.
- For experiment 7, why the procedure chosen for the counterconditioning was adopted from experiment 6 rather than experiment 5 which yielded more promising results?

Analysis
- In p. 7 the authors state "A 2 (Stimulus: neutral target related to positive vs. negative source) x 2 (Training: Extinction vs. Acquisition-only) mixed design was employed with the first factor measured within and the second measured between participants." Yet later, when reporting the Self-report ratings they state they indicate using "A one way ANOVA on target stimulus ratings (T1 and T2) with Training Condition as a between participants factor" - is it actually a mixed design, or is the DV is the difference between T1 and T2 (in that case there is no point in running ANOVA rather than a t-test on the two groups; this is also relevant for the IAT analysis). Please clarify this point.
- With regard to the logistic regression analysis, it would helpful if the model would be specified. Also, to determine whether there is a main effect of training a model comparison should be employed (between a model with and model without this factor).
- The meta analysis for the IR is highly informing. However, for the EOC the meta analysis is flawed as in some experiments extinction and counterconditioning were simply additional training sessions for the EOC contingencies.

Results
- Experiment 3, p.20 - "Intentions emerged in the expected direction in both the acquisition-only… and extinction conditions". This is somewhat misleading as the expected direction in this experiment for the OEC following extinction is different from following the acquisition.
- Experiment 4. p. 21 - "Each trial began with the presentation of a stimulus (T1, O1, T2, O2) for 1500ms (each stimulus was presented five times per block). " The word began is a bit misleading here as each trial includes only the presentation of a single stimulus.
- Experiment 7 - please indicate how many participants required a fifth block and what was the overall success rate.
- Experiment 7 - It is not entirely clear whether on extinction and counterconditioning the addition of a fifth block was dependent on the same condition as in training (please indicate explicitly in the text).
- Experiment 7 - please indicate when throughout the experiment you integrated the matching to sample (MTS) task.
- Experiment 7, p. 31 under Self-report ratings (intersecting regularities) - it seems that the degrees of freedom in the F-test are incorrect. Please check also the other reports of degrees of freedom in the results of this experiment as I suspect there other mistakes.

General Discussion:
- With regard to the theoretical discussion about the implications of this study, have the authors considered the option that the outcome becomes a secondary reinforcer upon acquiring the positive/negative value and thereby reinforce the target (neutral) stimulus? Would such case be reconciled with the potential theoretical implications discussed in the text or would use as an alternative explanation?
- The issue of the additional left/right side (and hand) intersection must be referred to in the general discussion, especially when referring to the ineffectiveness of the extinction.
- p. 35 - "evaluations… were reduced by counterconditioning-like procedures." In practice only one procedure yield a clear counterconditiong effect.
- p. 37 - "In line with earlier findings (e.g., Pavlov, 1927; Baeyens et al., 1988), our results are difficult to reconcile with associative models such as the Rescorla-Wagner model (Rescorla & Wagner, 1972; see also McCloskey & Cohen, 1989), which allow associations to weaken when contingencies no longer hold." When stating this I think the authors should make the distinction

that in the models mentioned the valenced part (the US) is the one traditionally removed from the contingency.
- p.38 - “The fact that a variety of extinction-like tasks did not reduce the magnitude of IR effects (Experiments 1, 2, and 4) can be explained by associative models only if it is assumed that the S1-O1 and T1-O1 associations are not weakened by the S1 and T1 presentations during the extinction phase.” But is it not the case that the very same explanation is contrasted in experiment 3?
- pp. 38-39 - “Resistance to extinction could also be accounted for on the basis of comparator-type models (e.g., Miller & Matzel, 1988) if one assumes that, unlike most other types of learned behavior, learned preferences do not depend on the output of a comparator mechanism, but directly reflect the strength of individual associations (e.g., De Houwer, 1998). ” I find the notion about comparator models here somewhat self-contradictory or unclear, please explain/elaborate.
- p.39 - “The results of Experiments 1-4 suggest that the latter inferred proposition may be maintained even when the premises of the inference (i.e., the propositions about the intersecting contingencies) no longer hold. ” It seems to me that this statement does not hold for experiment 3.
- A theoretical account for (or an attempt to explain) the success of extinction in experiment 3 compared to 1 and 2 could be insightful. Same goes for the success of counterconditioning in experiment 5 compared to 6 and 7.
- p. 43 “It would be worth examining the long-term effects and context dependency of counterconditioning via IR in order to determine if these changes in evaluation are stable across time and context.” Note that this was already suggested earlier in the discussion. For clarity you can just add “as mentioned before” or something of that sort.
- p. 44. “We also consistently manipulated parameters of the extinction and counterconditioning tasks from one study to the next which (a) may have made the task increasingly taxing on participants ”. Can you please elaborate on that?

Conclusion (p. 45):
- I think that in light of the variety of methods used and results obtained that the conclusion should be conveyed in a less deterministic manner with regard to the potential ability of exitinction and counterconditioning to change the evaluations formed through IR.
- The authors indicate that the “Same evaluations can be counterconditioned by… or (to a lesser extent) by reconfiguring the intersection”. However, when tested separately “reconfiguring the intersection” did not significantly change evaluations so this may draw confusion.

Suggestions for communicating purposes and to increase clarity:
- I suggest to include a figure illustrating the procedure/s, as common in papers reporting human behavioral experiments.
- As this experiment involves multiple experiments with multiple dependent variables for each experiment I would highly recommend to put together all the main results in some kind of a visualized table or plot indicating where significant results were found. One or a few barplots summarizing the results are also an option.
- The exclusions throughout the experiments are based on the same criteria, thus it is possible to add a table of exclusions by criterion for all the experiments and shorten the recurrent detailed description of them throughout the text.
- Keep using the specific keyboard key names as constantly associated with the same positive/negative stimuli throughout the text gives the wrong impression that this factor not counterbalanced (although it is stated that it was).

Please add relevant citations in the following cases:
- When mentioning preconditioning in the introduction.
- In p.35 for this statement: “This finding parallels result in evaluative conditioning studies in which the impact of counterconditioning procedures has also proven to be more robust than that of extinction procedures.”
- In p.38 for this statement: “Whereas many of these theoretical conclusions are supported not only by our findings but also by previous studies showing a lack of extinction of evaluative conditioning”.

Author's Response to Decision Letter for (RSOS-192085.R0)

See Appendix A.

Decision letter (RSOS-192085.R1)

We hope you are keeping well at this difficult and unusual time. We continue to value your support of the journal in these challenging circumstances. If Royal Society Open Science can assist you at all, please don't hesitate to let us know at the email address below.

Dear Dr Hughes,

It is a pleasure to accept your manuscript entitled "The Influence of Extinction and Counterconditioning Procedures on Operant Evaluative Conditioning and Intersecting Regularity Effects" in its current form for publication in Royal Society Open Science.

You can expect to receive a proof of your article in the near future. Please contact the editorial office (openscience_proofs@royalsociety.org) and the production office (openscience@royalsociety.org) to let us know if you are likely to be away from e-mail contact -- if you are going to be away, please nominate a co-author (if available) to manage the proofing process, and ensure they are copied into your email to the journal.

Due to rapid publication and an extremely tight schedule, if comments are not received, your paper may experience a delay in publication.

Please see the Royal Society Publishing guidance on how you may share your accepted author manuscript at https://royalsociety.org/journals/ethics-policies/media-embargo/.

Thank you for your fine contribution. On behalf of the Editors of Royal Society Open Science, we look forward to your continued contributions to the Journal.

Best regards,
Lianne Parkhouse
Editorial Coordinator
Royal Society Open Science
openscience@royalsociety.org

on behalf of Dr Joydeep Bhattacharya (Associate Editor) and Essi Viding (Subject Editor)
openscience@royalsociety.org

Follow Royal Society Publishing on Twitter: @RSocPublishing
Follow Royal Society Publishing on Facebook:
https://www.facebook.com/RoyalSocietyPublishing.FanPage/
Read Royal Society Publishing's blog: https://blogs.royalsociety.org/publishing/

Appendix A

Dear Dr Hughes

On behalf of the Editors, I am pleased to inform you that your Manuscript RSOS-192085 entitled "The Influence of Extinction and Counterconditioning on Operant Evaluative Conditioning and Intersecting Regularity Effects" has been accepted for publication in Royal Society Open Science subject to minor revision in accordance with the referee suggestions. Please find the referees' comments at the end of this email.

The reviewers and handling editors have recommended publication, but also suggest some minor revisions to your manuscript. Therefore, I invite you to respond to the comments and revise your manuscript.

• Ethics statement
If your study uses humans or animals please include details of the ethical approval received, including the name of the committee that granted approval. For human studies please also detail whether informed consent was obtained. For field studies on animals please include details of all permissions, licences and/or approvals granted to carry out the fieldwork.

• Data accessibility
It is a condition of publication that all supporting data are made available either as supplementary information or preferably in a suitable permanent repository. The data accessibility section should state where the article's supporting data can be accessed. This section should also include details, where possible of where to access other relevant research materials such as statistical tools, protocols, software etc can be accessed. If the data has been deposited in an external repository this section should list the database, accession number and link to the DOI for all data from the article that has been made publicly available. Data sets that have been deposited in an external repository and have a DOI should also be appropriately cited in the manuscript and included in the reference list.

If you wish to submit your supporting data or code to Dryad (http://datadryad.org/), or modify your current submission to dryad, please use the following link:
http://datadryad.org/submit?journalID=RSOS&manu=RSOS-192085

• Competing interests
Please declare any financial or non-financial competing interests, or state that you have no competing interests.
• Authors' contributions
All submissions, other than those with a single author, must include an Authors' Contributions section which individually lists the specific contribution of each author. The list of Authors should meet all of the following criteria; 1) substantial contributions to conception and design, or acquisition of data, or analysis and interpretation of data; 2) drafting the article or revising it critically for important intellectual content; and 3) final approval of the version to be published.

All contributors who do not meet all of these criteria should be included in the acknowledgements.

We suggest the following format:
AB carried out the molecular lab work, participated in data analysis, carried out sequence alignments, participated in the design of the study and drafted the manuscript; CD carried out the statistical analyses; EF collected field data; GH conceived of the study, designed the study, coordinated the study

and helped draft the manuscript. All authors gave final approval for publication.

• Acknowledgements
Please acknowledge anyone who contributed to the study but did not meet the authorship criteria.

• Funding statement
Please list the source of funding for each author.

Please ensure you have prepared your revision in accordance with the guidance at https://royalsociety.org/journals/authors/author-guidelines/ -- please note that we cannot publish your manuscript without the end statements. We have included a screenshot example of the end statements for reference. If you feel that a given heading is not relevant to your paper, please nevertheless include the heading and explicitly state that it is not relevant to your work.

Because the schedule for publication is very tight, it is a condition of publication that you submit the revised version of your manuscript before 06-Mar-2020. Please note that the revision deadline will expire at 00.00am on this date. If you do not think you will be able to meet this date please let me know immediately.

To revise your manuscript, log into https://mc.manuscriptcentral.com/rsos and enter your Author Centre, where you will find your manuscript title listed under "Manuscripts with Decisions". Under "Actions," click on "Create a Revision." You will be unable to make your revisions on the originally submitted version of the manuscript. Instead, revise your manuscript and upload a new version through your Author Centre.

When submitting your revised manuscript, you will be able to respond to the comments made by the referees and upload a file "Response to Referees" in "Section 6 - File Upload". You can use this to document any changes you make to the original manuscript. In order to expedite the processing of the revised manuscript, please be as specific as possible in your response to the referees. We strongly recommend uploading two versions of your revised manuscript:

1) Identifying all the changes that have been made (for instance, in coloured highlight, in bold text, or tracked changes);
2) A 'clean' version of the new manuscript that incorporates the changes made, but does not highlight them.

When uploading your revised files please make sure that you have:

1) A text file of the manuscript (tex, txt, rtf, docx or doc), references, tables (including captions) and figure captions. Do not upload a PDF as your "Main Document";
2) A separate electronic file of each figure (EPS or print-quality PDF preferred (either format should be produced directly from original creation package), or original software format);
3) Included a 100 word media summary of your paper when requested at submission. Please ensure you have entered correct contact details (email, institution and telephone) in your user account;
4) Included the raw data to support the claims made in your paper. You can either include your data as electronic supplementary material or upload to a repository and include the relevant doi within your manuscript. Make sure it is clear in your data accessibility statement how the data can be accessed;
5) All supplementary materials accompanying an accepted article will be treated as in their final form.

Note that the Royal Society will neither edit nor typeset supplementary material and it will be hosted as provided. Please ensure that the supplementary material includes the paper details where possible (authors, article title, journal name).

Supplementary files will be published alongside the paper on the journal website and posted on the online figshare repository (https://rs.figshare.com/). The heading and legend provided for each supplementary file during the submission process will be used to create the figshare page, so please ensure these are accurate and informative so that your files can be found in searches. Files on figshare will be made available approximately one week before the accompanying article so that the supplementary material can be attributed a unique DOI.

Please note that Royal Society Open Science charge article processing charges for all new submissions that are accepted for publication. Charges will also apply to papers transferred to Royal Society Open Science from other Royal Society Publishing journals, as well as papers submitted as part of our collaboration with the Royal Society of Chemistry (https://royalsocietypublishing.org/rsos/chemistry).

If your manuscript is newly submitted and subsequently accepted for publication, you will be asked to pay the article processing charge, unless you request a waiver and this is approved by Royal Society Publishing. You can find out more about the charges at. Should you have any queries, please contact openscience@royalsociety.org.

Once again, thank you for submitting your manuscript to Royal Society Open Science and I look forward to receiving your revision. If you have any questions at all, please do not hesitate to get in touch.

Kind regards,

Anita Kristiansen
Editorial Coordinator

Royal Society Open Science
openscience@royalsociety.org

on behalf of Dr Joydeep Bhattacharya (Associate Editor) and Essi Viding (Subject Editor)
openscience@royalsociety.org

Associate Editor (Dr Joydeep Bhattacharya):
Comments to the Author:
All reviewers found merits in your revised version, and especially, the experimental rigor has been highlighted. The reviewers and I appreciate the extensive amount of work you've put in this submitted work. However, there are still some important issues (especially a potential confound as mentioned by the Reviewer-3) that need to be clarified; further, Reviewer-3 has also made some useful suggestions for your considerations for improving the clarity. I am looking forward to receiving a revised final version.

Authors: We thank the Editor and Reviewers 1-3 for their continued input. Based on this feedback we went back to the paper and revised and condensed. Moreover, we updated the processing and analyses in R so that our data treatment is now fully transparent and reproducible. In addition to these changes we have also respond to each of the points highlighted by the reviewers below.

We believe that this version of the paper is significantly stronger than our previous submission.

Reviewer comments to Author:
Reviewer: 1

Comments to the Author(s)
I found all of my concerns adequately addressed by the authors and believe that this makes a fine paper. Due to their clarifications, I now find the intersecting regularities perspective quite inspiring.

Authors: We thank Reviewer 1 for their feedback and kind words.

Reviewer 2: In their manuscript "The Influence of Extinction and Counterconditioning on Operant Evaluative Conditioning and Intersecting Regularity Effects", Hughes and colleagues discuss a large set of experiments in which evaluation changes are acquired via intersecting regularities. These changes are then subjected to various systematically varied interventions designed to reverse them in order to test determinants of their robustness. Coming in as a new reviewer on a revision is always a challenge. I have studied the comments by the previous reviewers and compared them to the manuscript's current form. In my view, the authors have successfully addressed the issues raised in the previous reviews. For my own part, I find the paper examines a scientifically relevant question with a strong empirical basis. While this kind of nitty-gritty research isn't always the most glamourous, it is important to carefully dissect the operating circumstances of these processes.

Authors: We thank Reviewer 2 for their feedback and kind words.

Reviewer 2: Therefore, I only have one concern before I can recommend this manuscript for acceptance, namely that I don't understand how the authors get 47 degrees of freedom from a 51 person sample in their pretest (p.18 of the pdf). This is likely a simple error to correct.

Authors: We thank the reviewer for pointing this out to us. We collected data from 51 participants but only 47 provided us with complete data. We therefore only ran our analyses on the latter. We have now revised the manuscript to clarify this point (see changes on p. 9).

Reviewer 2: p.37 "beyond the constrains" should be "beyond the constraints"

Authors: This sentence is no longer in the revised manuscript.

Reviewer 2: p.39 "account for the lack of extinction in our studies be it that forces them" – this makes no grammatical sense as written

Authors: This sentence is no longer in the revised manuscript.

Reviewer 2: Finally, the sheer amount of analyses over so many studies and dependent variables can be quite tiring as a reader – as a quality-of-life improvement, I think summarizing non-significant results in the main text in a simple sentence and presenting the full analyses in an appendix might be useful.

Authors: Based on Reviewer 2's suggestions, analyses have been condensed in the ways suggested here (see revisions to the Results sections). Non-critical analyses have also been moved to the Supplementary Materials section.

Beyond these few issues, I recommend the manuscript for publication.
Signed
Anand Krishna

Reviewer 3: This study has widely examined the capacity of several variants of (analogues to) extinction and counterconditioning to diminish or reverse the evaluation formed via intersecting regularities. The paper is mostly clear and easy to read. I praise the authors for placing all the materials related to the study in an organized and comprehensive manner, which is helps to promote open science.

It is evident that a considerable amount of work has been invested in this work. Multiple experiments were designed and executed to tackle the research question. This resulted in a relatively comprehensive work that helps to extend our knowledge about associative learning and in particular about the malleability of conditioned evaluations formed through evaluative (operant) conditioning and an extension of it termed intersecting regularities. Notably, the authors perform meta-analyses on the results of the seven experiments conducted in this study which further inform the reader.

Nevertheless, this work includes a few methodological problems which should be taken in consideration and a few clarifications are needed with regard to several parts of this work.
I raise here a few main issues and some minor issues/corrections/clarifications needed as well as a few suggestions to potentially better communicate this work. I start with a few general notes and then my comments mostly follow to different parts of the manuscript (where most but not all comments are minor).

Authors: We thank Reviewer 3 for their comments and respond to each below.

Reviewer 3: General comments:
- A substantial methodological problem in this study is the additional intersecting component of the side right or left (accompanied with the use of the right or left hand). This was recognized by the authors who committed an attempt to address it in experiment 7, which if anything, demonstrated the importance of this factor. This intersection was presented along experiments 1 to 6 during training and in experiments 1-3 and 6 also during extinction/counterconditioning (* but notably not in experiment 5, unlike what the authors indicated in p. 29). This is a serious confound that compel us to take the conclusions drawn from this work with a grain of salt and creates a special necessity to verify them in future work. In my view this point should be highlighted throughout the manuscript wherever appropriate (currently it is firstly mentioned in the description of experiment 7) and accordingly the conclusions should be described with caution and with a proportional amount of confidence. On a side

none, with regard to experiment 3, where extinction was effective, the presence of an additional intersection possibly suggests that the type of extinction procedure used in this case has high efficacy.

Authors: As the reviewer indicates, we acknowledge this issues as a potential confound in the studies. We now mention it in the introduction and devote a paragraph to it in the general discussion. That said, we still failed to observe extinction in Experiments 4 and 7 (where no such confound was present) and did observe counterconditioning in Experiment 5 (where it was partially present). As we now acknowledge in the general discussion, we believe that this factor may moderate extinction and counterconditioning but not fully explain the findings reported here (see changes on p.6, and 40-41).

Reviewer 3: Since the experiments were preformed on-line, special measures are required to ensure participants' engagement. The only exclusion criteria with regard to participants engagement were based on the IAT (except for experiment 7). Learning criteria for the acquisition and extinction/counterconditioning phases are required, or at least providing evidence that all the participants were engaged during these phases. Specifying mean and SD for accuracy and indicate whether there are outliers as well as testing that removing those outliers does not affect the results would be satisfying. Also, as this could not be done for experiment 4 (passive viewing of the neutral stimuli), this should be discussed as a potential cause for the lack of extinction.

Authors: In line with the reviewer's suggestion, we have made three changes to our manuscript. First, we now report performance metrics (accuracy means and SDs) for the acquisition, extinction, and counterconditioning groups in Experiments 1-7. Second, we indicate the percentage of participants who "passed" each phase of the learning task (i.e., responded with greater than 75% accuracy on the final block of a given phase). Third, we included a series of sensitivity analyses wherein outliers (participants who failed the learning task) were excluded and analyses rerun. These new additions make it clear that (a) accuracy was consistently high in each phase of each experiment, (b) that the majority of participants "passed" each phase of the learning task, and (c) that the conclusions drawn throughout the paper still hold whenever outliers are removed (see revised Results sections throughout).

Reviewer 3: Throughout the paper in general and the discussion in particular the results of experiment 3 are largely ignored.

Authors: We have rewritten the paper and revised the data processing and analyses in R so that they are fully transparent and reproducible. In the process of doing so we now speak to the nuances of each experiment (including Experiment 3), and more importantly, carry out a series of multi-level meta-analyses in order to answer our "top line" questions: is there evidence of evaluative learning *in general*, is this moderated by extinction or counterconditioning *in general*, and do these effects change when we exclude outliers? (see the revised manuscript).

Reviewer 3: Please make the pre-registration documents accessible as currently accessing them requires permission.

Authors: The pre-registration and all materials are now publically available at https://osf.io/u6vtz/

Reviewer 3: I tried to reproduce the results to gain a better understanding of some analyses but was unable to because there are only data files (or sav files with no log). I highly suggest to provide an easy way to reproduce your results (you can add the sass script you have used for instance).

Authors: Based on the reviewer's comment we went back and rewrote all data processing and analysis files in R (see https://osf.io/u6vtz/). In this way our processing and analyses are now fully transparent and reproducible.

Reviewer 3: Introduction
- Some more background information about operant evaluative conditioning will be useful.

Authors: additional information about operant evaluative conditioning has been added (see changes on p.7).

Reviewer 3: Although adopted from a previous study, the operant conditioning procedure used here is not trivial. Traditionally in operant conditioning a subject is exposed to a neutral stimulus and a response is chosen freely and is followed by a valenced stimulus and not vice vera. I think that a few words about why choosing this procedure and what role does the response play here would help orienting the reader.

Authors: We have now included a footnote acknowledging that operant evaluative conditioning can occur in multiple ways, and that the procedure developed in this paper is but one way of instantiating OEC effects (see footnote six on p.7).

Reviewer 3: p. 5 With regard to counterconditioning "During a first phase (acquisition) a contingency is established between two stimuli by pairing a neutral CS with a positive US. " Note that for clarity you should mention the opposite option of a negative US as it is referred in the subsequent lines.

Authors: This sentence has been revised as suggested (see changes on p.5).

Reviewer 3: p.5 "This work was not designed to differentiate between two competing cognitive theories" - it is not clear in this context what are the competing cognitive theories are you referring to.

Authors: This sentence is no longer in the revised manuscript.

Reviewer 3: The examples for extinction and counterconditioning are given in the form of classical conditioning whereas you this study is using operant conditioning. Therefore, I recommend to set these examples in the context of operant conditioning.

Authors: Given that (a) the vast majority of work on extinction and counterconditioning is carried out in the context of EC, and (b) these paradigms are most familiar to our intended audience, we decided to keep the examples as they were.

Reviewer 3: p.6 "We refer to this as an extinction-like procedure because, similar to extinction tasks in EC, it involves the removal of the environmental event that underlies the target evaluation (in this case the common element shared by regularities)". This is true, yet notably, extinction traditionally removes the valenced stimulus. I propose to indicate this important distinction.

Authors: We now highlight this point (see footnote 4 on p.5).

Reviewer 3: The IAT is not explained clearly enough (e..g, in the first two blocks there is only one item on each side and is not clear from text). Please edit accordingly and/or add a figure with illustration.

Authors: The description of the IAT procedure has been updated to improve clarity (see changes on p.14).

Reviewer 3: p. 7 "We added the IAT because it is assumed to capture more automatic instances of evaluation that can influence behavior in unique ways" unique ways is rather vague, I suggest to elaborate (or rephrase).

Authors: Sentence revised to avoid this issue (see changes on p.7):

“We added the IAT because it is assumed to capture more automatic instances of evaluation”

Reviewer 3: Is there a reason for the discrepancy in the error feedback duration between extinction and acquisition (3s vs 2s)?

Authors: We thank the reviewer for pointing this typo out to us. We went back and corrected the reporting of trial timings in this and all experiments (see revised manuscript).

Reviewer 3: p. 11 With regard to the behavioral intention task, it is not entirely clear which items/stimuli were compared and in which manner. Please describe the task more comprehensively (a figure illustrating the task is also an option).

Authors: The description of the behavioral intention task has been updated to aid clarity (see changes on p.15).

Reviewer 3: p. 12 Exploratory questions section - although exploratory, as the memory test was also conducted between the acquisition and the following extinction/counterconditioning phase, one may be concerned that it may affect the evaluation results. Therefore, please describe the test. Additionally, were the final memory test and the other exploratory measures conducted after recording the evaluative measures?

Authors: We have included a more comprehensive description of the testing phases as requested (see changes on p.15-16). The testing phases were administered after the acquisition and intervention phases, whereas the exploratory questions were asked after the evaluative measures were completed.

Reviewer 3: Experiment. 5 p. 24 - “In all cases these were similar to those reported in Experiments 1-5 unless otherwise stated. ” I believe this should be Experiments 1-4.

Authors: This sentence is no longer in the revised manuscript.

Reviewer 3: For experiment 7, why the procedure chosen for the counterconditioning was adopted from experiment 6 rather than experiment 5 which yielded more promising results?

Authors: Honestly, the justification for this choice was made several years ago and cannot be recalled.

Reviewer 3: Analysis - In p. 7 the authors state “A 2 (Stimulus: neutral target related to positive vs. negative source) x 2 (Training: Extinction vs. Acquisition-only) mixed design was employed with the first factor measured within and the second measured between participants.” Yet later, when reporting the Self-report ratings they state they indicate using “A one way ANOVA on target stimulus ratings (T1 and T2) with Training Condition as a between participants factor” - is it actually a mixed design, or is the DV is the difference between T1 and T2 (in that case there is no point in running ANOVA rather than a t-test on the two groups; this is also relevant for the IAT analysis). Please clarify this point.

Authors: These analyses are no longer carried out in the revised version of the paper (see revised Results section for new analyses).

Reviewer 3: With regard to the logistic regression analysis, it would helpful if the model would be specified. Also, to determine whether there is a main effect of training a model comparison should be employed (between a model with and model without this factor).

Authors: These analyses are no longer carried out in the revised version of the paper (see revised Results section for new analyses).

Reviewer 3: The meta analysis for the IR is highly informing. However, for the OEC the meta analysis is flawed as in some experiments extinction and counterconditioning were simply additional training sessions for the EOC contingencies.

Authors: These analyses are no longer carried out in the revised version of the paper (see revised Results section for new analyses).

Reviewer 3: Results - Experiment 3, p.20 - "Intentions emerged in the expected direction in both the acquisition-only… and extinction conditions". This is somewhat misleading as the expected direction in this experiment for the OEC following extinction is different from following the acquisition.

Authors: This sentence is no longer in the revised manuscript.

Reviewer 3: Experiment 4. p. 21 - "Each trial began with the presentation of a stimulus (T1, O1, T2, O2) for 1500ms (each stimulus was presented five times per block). " The word began is a bit misleading here as each trial includes only the presentation of a single stimulus.

Authors: Sentence revised as requested (see changes on p.10, 11, 23).

Reviewer 3: - Experiment 7 - It is not entirely clear whether on extinction and counterconditioning the addition of a fifth block was dependent on the same condition as in training (please indicate explicitly in the text).

Authors: Additional information is provided to clarify that the same criterion was used in acquisition as in extinction and counterconditioning (see changes on p.28-29).

Reviewer 3: - Experiment 7 - please indicate when throughout the experiment you integrated the matching to sample (MTS) task.

Authors: The MTS was always provided at the very end of the study. The method section has been updated to reflect this (see changes on p.29).

Reviewer 3: Experiment 7, p. 31 under Self-report ratings (intersecting regularities) - it seems that the degrees of freedom in the F-test are incorrect. Please check also the other reports of degrees of freedom in the results of this experiment as I suspect there other mistakes.

Authors: These analyses are no longer carried out in the revised version of the paper (see revised Results section for new analyses).

Reviewer 3: General Discussion: - With regard to the theoretical discussion about the implications of this study, have the authors considered the option that the outcome becomes a secondary reinforcer upon acquiring the positive/negative value and thereby reinforce the target (neutral) stimulus? Would such case be reconciled with the potential theoretical implications discussed in the text or would use as an alternative explanation?

Authors: Good point. It would indeed count as an alternative explanation. We have now added a discussion of this alternative account to the general discussion.

Reviewer 3: The issue of the additional left/right side (and hand) intersection must be referred to in the general discussion, especially when referring to the ineffectiveness of the extinction.
- p. 35 - "evaluations… were reduced by counterconditioning-like procedures." In practice only one procedure yield a clear counterconditioning effect.

Authors: We have included a section in the general discussion section that refers to the right/left hand intersection and how it may moderate the findings reported here (see changes on p. 40-41).

Reviewer 3: p. 37 - "In line with earlier findings (e.g., Pavlov, 1927; Baeyens et al., 1988), our results are difficult to reconcile with associative models such as the Rescorla-Wagner model (Rescorla & Wagner, 1972; see also McCloskey & Cohen, 1989), which allow associations to weaken when contingencies no longer hold." When stating this I think the authors should make the distinction that in the models mentioned the valenced part (the US) is the one traditionally removed from the contingency.

Authors: In-line with the reviewer's suggestion we now mention this in the general discussion (see footnote 12 on p.41).

Reviewer 3: p.38 - "The fact that a variety of extinction-like tasks did not reduce the magnitude of IR effects (Experiments 1, 2, and 4) can be explained by associative models only if it is assumed that the S1-O1 and T1-O1 associations are not weakened by the S1 and T1 presentations during the extinction phase." But is it not the case that the very same explanation is contrasted in experiment 3?

Authors: This sentence has been revised in light of new analyses that were conducted. Extinction effects were no longer observed in Experiment 3.

Reviewer 3: pp. 38-39 - "Resistance to extinction could also be accounted for on the basis of comparator-type models (e.g., Miller & Matzel, 1988) if one assumes that, unlike most other types of learned behavior, learned preferences do not depend on the output of a comparator mechanism, but directly reflect the strength of individual associations (e.g., De Houwer, 1998). " I find the notion about comparator models here somewhat self-contradictory or unclear, please explain/elaborate.

Authors: It is indeed difficult to understand this statement without additional information about comparator models. Instead of adding a (necessarily lengthy) description of the comparator model, we now convey the core idea behind how this model (as well as the model of Baeyens) might account for the absence of extinction effects.

Reviewer 3: p.39 - "The results of Experiments 1-4 suggest that the latter inferred proposition may be maintained even when the premises of the inference (i.e., the propositions about the intersecting contingencies) no longer hold. " It seems to me that this statement does not hold for experiment 3.

Authors: Extinction effects were no longer observed in Experiment 3 after novel analyses.

Reviewer 3: A theoretical account for (or an attempt to explain) the success of extinction in experiment 3 compared to 1 and 2 could be insightful. Same goes for the success of counterconditioning in experiment 5 compared to 6 and 7.

Authors: Without additional studies, it is difficult to be sure how reliable the observed extinction and counterconditioning effects. Hence, we think it is premature to discuss these preliminary findings in terms of different theoretical accounts. Moreover, we are wary of adding speculative discussions to a paper that is already quite long.

Reviewer 3: p. 43 "It would be worth examining the long-term effects and context dependency of counterconditioning via IR in order to determine if these changes in evaluation are stable across time and context." Note that this was already suggested earlier in the discussion. For clarity you can just add "as mentioned before" or something of that sort.

Authors: This sentence has been deleted as suggested.

Reviewer 3: p. 44. "We also consistently manipulated parameters of the extinction and counterconditioning tasks from one study to the next which (a) may have made the task increasingly taxing on participants ". Can you please elaborate on that?

Authors: This sentence was indeed ambiguous and unnecessary and was therefore deleted.

Reviewer 3: Conclusion (p. 45): - I think that in light of the variety of methods used and results obtained that the conclusion should be conveyed in a less deterministic manner with regard to the potential ability of exitinction and counterconditioning to change the evaluations formed through IR.

Authors: The conclusion section has been revised and qualified with more nuance (see changes on p.41).

Reviewer 3: - The authors indicate that the "Same evaluations can be counterconditioned by… or (to a lesser extent) by reconfiguring the intersection". However, when tested separately "reconfiguring the intersection" did not significantly change evaluations so this may draw confusion.

Authors: Indeed. We therefore deleted this sentence.

Reviewer 3: Suggestions for communicating purposes and to increase clarity: - I suggest to include a figure illustrating the procedure/s, as common in papers reporting human behavioral experiments.

Authors: Based on this suggestion we have included a Figure (2) that highlights the procedures used in Experiments 1-7 (see changes on p.13).

Reviewer 3: - As this experiment involves multiple experiments with multiple dependent variables for each experiment I would highly recommend to put together all the main results in some kind of a visualized table or plot indicating where significant results were found. One or a few barplots summarizing the results are also an option.

Authors: See Figures 3 and 4 for forest plots of the effects from all seven studies for the IR and OEC effects.

Reviewer 3: - The exclusions throughout the experiments are based on the same criteria, thus it is possible to add a table of exclusions by criterion for all the experiments and shorten the recurrent detailed description of them throughout the text.

Authors: The sections of exclusion have been condensed in the revised version of the paper based on this suggestion (see Results sections throughout the paper).

Reviewer 3: - Keep using the specific keyboard key names as constantly associated with the same positive/negative stimuli throughout the text gives the wrong impression that this factor not counterbalanced (although it is stated that it was).

Authors: We revised the paper so that we make reference to R1, R2, R3, and R4, instead of the actual keys (as key assignment was counterbalanced as the reviewer correctly points out).

Reviewer 3: Please add relevant citations in the following cases:
- When mentioning preconditioning in the introduction.
- In p.35 for this statement: "This finding parallels result in evaluative conditioning studies in which the impact of counterconditioning procedures has also proven to be more robust than that of extinction procedures."
- In p.38 for this statement: "Whereas many of these theoretical conclusions are supported not only by our findings but also by previous studies showing a lack of extinction of evaluative conditioning".

Authors: Citations have been added in cases where the sentence and claim is still part of the paper.
